# Analysis of knockout mutants reveals non-redundant functions of poly(ADP-ribose)polymerase isoforms in Arabidopsis

**DOI:** 10.1007/s11103-015-0363-5

**Published:** 2015-10-01

**Authors:** Phuong Anh Pham, Vanessa Wahl, Takayuki Tohge, Laise Rosado de Souza, Youjun Zhang, Phuc Thi Do, Justyna J. Olas, Mark Stitt, Wagner L. Araújo, Alisdair R. Fernie

**Affiliations:** Max-Planck-Institute of Molecular Plant Physiology, Am Mühlenberg 1, 14476 Potsdam-Golm, Germany; Faculty of Biology, VNU University of Science, Vietnam National University, Hanoi, Hanoi, Vietnam; Max-Planck-Partner Group at the Departamento de Biologia Vegetal, Universidade Federal de Viçosa, Viçosa, MG 36570-900 Brazil

**Keywords:** *Arabidopsis thaliana*, Central carbon metabolism, T-DNA mutants, Metabolite profiling, NAD(P)(H) metabolism, Poly(ADP-ribose)polymerase, Root

## Abstract

The enzyme poly(ADP-ribose)polymerase (PARP) has a dual function being involved both in the poly(ADP-ribosyl)ation and being a constituent of the NAD^+^ salvage pathway. To date most studies, both in plant and non-plant systems, have focused on the signaling role of *PARP* in poly(ADP-ribosyl)ation rather than any role that can be ascribed to its metabolic function. In order to address this question we here used a combination of expression, transcript and protein localization studies of all three *PARP* isoforms of Arabidopsis alongside physiological analysis of the corresponding mutants. Our analyses indicated that whilst all isoforms of PARP were localized to the nucleus they are also present in non-nuclear locations with *parp1* and *parp3* also localised in the cytosol, and *parp2* also present in the mitochondria. We next isolated and characterized insertional knockout mutants of all three isoforms confirming a complete knockout in the full length transcript levels of the target genes as well as a reduced total leaf NAD hydrolase activity in the two isoforms (*PARP1*, *PARP2*) that are highly expressed in leaves. Physiological evaluation of the mutant lines revealed that they displayed distinctive metabolic and root growth characteristics albeit unaltered leaf morphology under optimal growth conditions. We therefore conclude that the *PARP* isoforms play non-redundant non-nuclear metabolic roles and that their function is highly important in rapidly growing tissues such as the shoot apical meristem, roots and seeds.

**Electronic supplementary material:**

The online version of this article (doi:10.1007/s11103-015-0363-5) contains supplementary material, which is available to authorized users.

## Introduction

Nicotinamide adenine dinucleotide (NAD^+^) and its derivatives play a critical role in various metabolic events for maintaining energy homeostasis in living organisms. Given the fact that the reactions utilizing NAD^+^ are manifold it is unsurprising that redundancy exists in its pathways of biosynthesis with different routes to the same end, namely the *de novo* pathway and the salvage pathway (Hashida et al. 2009; Pétriacq et al. 2012). The *de novo* pathway comprises of five enzyme catalyzed reactions beginning with the conversion of aspartate to quinolate via the concerted action of aspartate oxidase and quinolate synthase (Schippers et al. 2008), through quinolinate phosphoribosyltransferase and nicotinate (and/or nicotinamide) mononucleotide adenylyltransferase prior to the conversion of nicotinate adenine dinucleotide (NaAD) to NAD^+^ by NAD synthase (NADS) (Hashida et al. 2009). Concomitantly, the four-step salvage pathway promotes the degradation of NAD^+^ to nicotinamide (NaM) as a result of (cyclic)ADP-ribose generation (via ADPR-cyclase), poly(ADP-ribosyl)ation (by PARP) or alternatively during the course of protein deacetylation (by SRT2) (Hunt et al. 2004; König et al. 2014). NaM is then deamidated to nicotinic acid (Na), which is transferred onto 5′-phosphoribosyl-1-pyrophosphate (PRPP) via nicotinate phosphoribosyl-transferase (NAPRT). The resultant NaMN subsequently acts as substrate for nicotinamide mononucleotide adenylyltransferease (NaMNAT), a *de novo* pathway enzyme, to produce nicotinate adenine dinucleotide (NaAD), which is then recycled to NAD^+^ (Hunt et al. 2004), thus closing the cycle. Intriguingly, this recycling system in *Arabidopsis* is quite different from the well-known two-step salvage pathway in mammals, where NAD^+^ is a product of nicotinamide mononucleotide (NMN) via an adenylylation reaction (Hashida et al. 2009).

NAD^+^-biosynthesis is crucial in multiple metabolic events and therefore a continuous synthesis of NAD^+^ is of vital importance to all cells. As mentioned previously in (Belenky et al. 2007), not only does NAD^+^ act as an coenzyme, whose concentration must be maintained to sustain basic cellular redox and energy metabolism, but also as substrate for NaM producing enzymes, particularly ADP-ribose transferase also known as poly(ADP-ribose)polymerase (PARP). The latter is the key enzyme in one of the major NAD^+^-consuming process, the poly(ADP-ribosyl)ation. This covalent post-translational protein modification, which utilizes NAD^+^ as substrate for NaM synthesis, has caught notable attention over decades in animal research (Briggs and Bent 2011; Bürkle 2001). In animals, poly(ADP-ribosyl)ation participates in several cellular processes such as DNA-repair, DNA-replication, regulation of cell cycle and in maintaining genomic stability (Sallmann et al. 2000; Trucco et al. 1998). During the reaction catalyzed by this enzyme branched ADP-ribose polymers are formed and attached to glutamate residues on specific protein receptors (Adams-Phillips et al. 2010; Hunt and Gray 2009; Hunt et al. 2004). The modification is reversed by poly(ADP-ribose) glycohydrolase (PARG), which hydrolyzes poly(ADP-ribose) polymers to form free ADP-riboses (Briggs and Bent 2011; Meyer et al. 2006). Due to the fact that poly(ADP-ribosyl)ation plays a vital role in numerous cellular processes and also affect heart attack, ischemia, Alzheimer and sensitivity of therapeutic cancer treatment reagents (Andrabi et al. 2006; Briggs and Bent 2011; Meyer et al. 2006), the development of PARP inhibitors has been a priority for pharmaceutical companies (Briggs and Bent 2011; Heeres and Hergenrother 2007).

By contrast, very few reports in plant systems have been published to date (see Briggs and Bent 2011; Jia et al. 2013; Pétriacq et al. 2012; Schulz et al. 2012). The first reports on the function of *PARP* in plants appeared in the late 70s (Payne and Bal 1976; Whitby and Whish 1977), where poly(ADP-ribosyl)ation was detected in nuclei of both germinated and non-germinated *Allium cepa* seeds as well as in isolated nuclei from root tips of *Triticum aestivum*. Only relatively recently, however, was the role of *PARP* during biotic and abiotic stress responses revealed (Amor et al. 1998; de Block et al. 2005; Vanderauwera et al. 2007). Furthermore, molecular genetic approaches were only attempted very recently (Jia et al. 2013; Rissel et al. 2014; Schulz et al. 2012). These studies suggested the participation of *PARP* in stress response, activation of non-homologous end-joining repair mechanism and seed development. It is important to note however that the first study via use of non-specific chemical and genetic inhibition did not dissect the roles of the specific isoforms. Furthermore, a detailed basic molecular characterization of the expression and localization of the independent isoforms is currently lacking. Here we, therefore, investigated expression and localization for the three Arabidopsis isoforms of *PARP*. In addition we physiologically characterized *parp1*, *parp2* and *parp3* mutants at the level of germination rate, root growth, photosynthetic performance, reproductive development and shoot. The results presented are discussed in terms of the consequences of the mutations on NAD^+^ metabolism, metabolic fluxes and whole plant vegetative and reproductive phenotypes suggesting potential isoform-specific non-nuclear roles for the *PARP*s in *Arabidopsis thaliana*.

## Results

### Expression patterns of PARP1, PARP2 and PARP3 in arabidopsis

Despite the fact that previous studies have focused on the biological function of *PARP* in Arabidopsis (Jia et al. 2013; Rissel et al. 2014; Schulz et al. 2012), surprisingly they did not carry out basic expression analysis of the three *PARP* genes encoded in the Arabidopsis genome (indeed this information is currently available only for *PARP3*). Scanning the Bio-Analytic Resource for Plant Biology website (BAR, bar.utoronto.ca, Toufighi et al. 2005), suggests high expression of *PARP1* and *PARP2* in the shoot apex, young siliques between stage three and five, and closed and open flowers. Moreover these two genes also appear to be expressed in dry seeds, young seedlings and late seedlings. By contrast, *PARP3* is suggested to be expressed only in dry seeds and seeds of mature siliques (Jia et al. 2013; Rissel et al. 2014; Schulz et al. 2012, 2014). Our own data in leaves, roots and seeds were very much in support of this with *PARP3* barely being expressed in leaves and roots but massively so in seeds with the other isoforms displaying contrasting expression patterns (Table 1).

**Table 1:** Relative expression analysis of NAD-biosynthesis genes in wild type Col-0

	Leaves	SE	Roots	SE	Seeds	SE
PARP1	25.1105	11.6140	0.221	0.109	0.096	0.029
PARP2	34.0041	15.4816	0.520	0.237	1.384	0.396
PARP3	0.0662	0.0242	0.001	0.000	3991.535	2634.160

Leaves and roots from 3-week-old Col-0 plants grown in half strength MS media plus 1 % sucrose plates under long day (16 h photoperiod) and dry seeds were used for RNA isolation and cDNA synthesis. Quantitative real time PCR was performed to determine the expression level of the following genes: *PARP1, PARP2 * and *PARP3; ACT2* was used as a housekeeping gene. Values are expressed as relative expression values ± SE of four biological replicates, normalized by the housekeeping gene

**Fig. 1:**
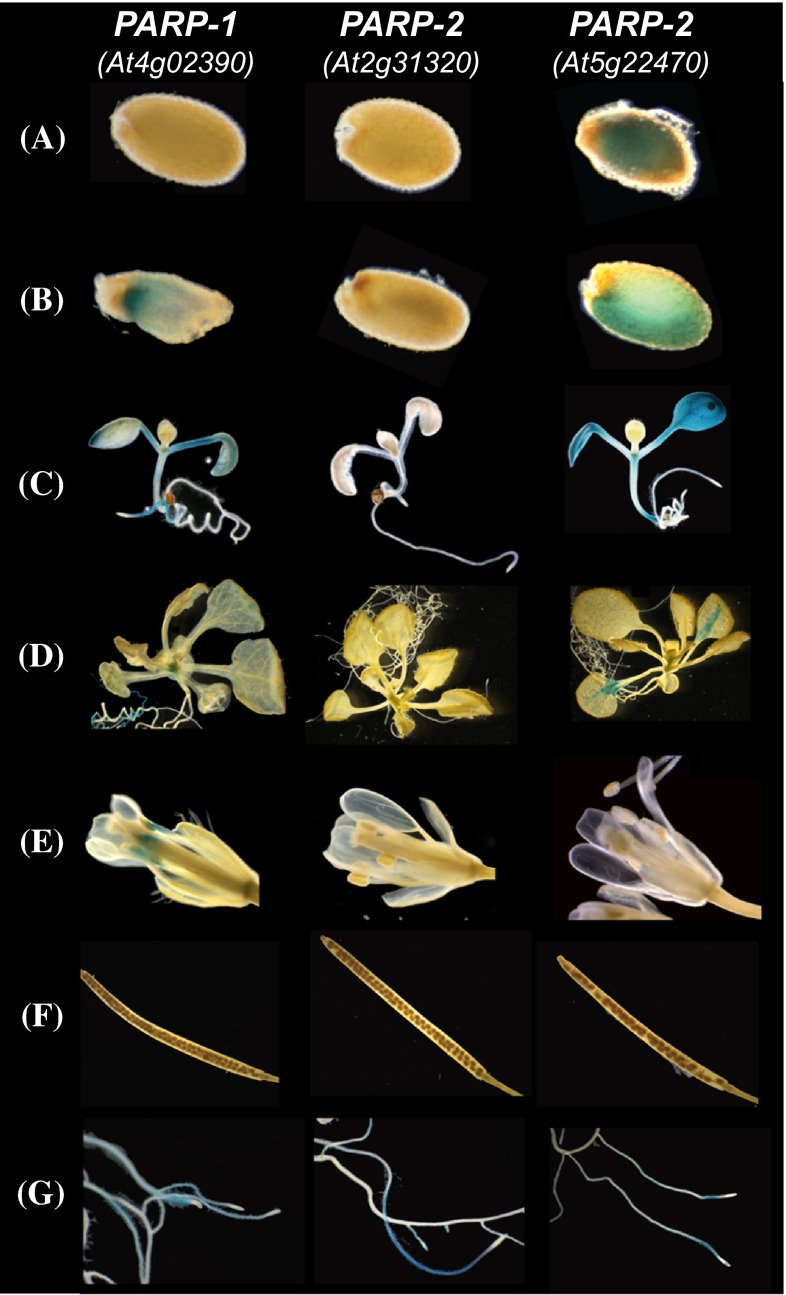
Histochemical staining of pPARP::GUS fused plants grown in long day (16 h light period) showing GUS-reporter activity in dry seed (*a*), imbibed seed (*b*), young seedling (*c*), 21-day-old vegetative rosette (*d*), open flower (*e*), silique (*f*) and root (*g*)

**Fig. 2:**
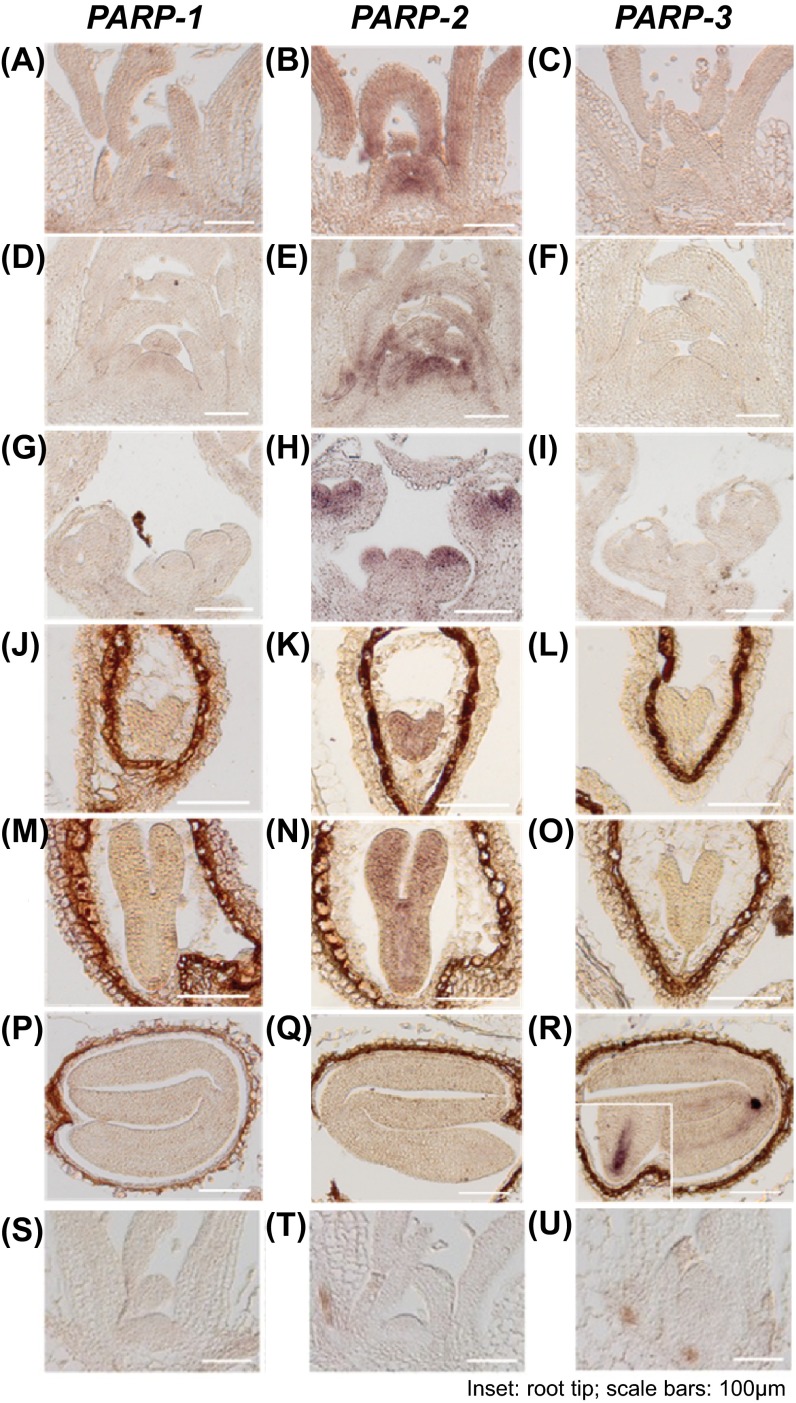
RNA in situ hybridization using specific probes for the *PARP* genes on longitudinal sections through vegetative apices (**a**–**c** long day; **d**–**f** short day), inflorescence apices (**g**–**i**), embryo at various stages (**j**–**l** heart stage; **m**–**o** torpedo stage; **p**–**r** U stage) and sense control in long day vegetative apices (**s**–**u**)

In order to investigate organ and cell specificity expression in more detail, the *PARP1, PARP2 and PARP3* 5′ upstream regions (2360 base pair, 2047 base pair and 1833 base pair, respectively) were fused to the *β*-glucuronidase (*GUS*) gene. In dry seeds *GUS* expression was only visible in plants bearing the *GUS* gene under the control of the *PARP3* 5′ upstream region (Fig. 1a), however in imbibed seeds and young seedlings staining was visible when *GUS* expression was regulated by the *PARP1* and *PARP3* promoters (Fig. 1b, c). In addition, in older seedlings (21 day old), *GUS* staining was highest in pPARP1::GUS and pPARP3::GUS lines (Fig. 1d). According to our *GUS* lines *PARP1* was the only isoform highly expressed in the stamen of the open flower (Fig. 1e). None of the isoforms were expressed in siliques (Fig. 1f), whilst *PARP1, PARP2 and PARP3* were all expressed in roots (Fig. 1g). These results thus largely mirror those described found on the Bio-Analytic Resource for Plant Biology website (BAR, bar.utoronto.ca, Toufighi et al. 2005), however, since *GUS* reporter lines prone to lacking regulatory elements in the 5′ upstream region fused to the reporter gene, we wanted to compare the expression pattern with RNA in situ hybridizations on various tissue sections (Fig. 2). While *PARP1* was only present at very low concentrations at the shoot apex of vegetative meristems from long day and short day grown plants and in heart, torpedo and U-stage embryos (Fig. 2a, d, g, j, m, p), *PARP*2 transcripts were highly abundant in vegetative as well as inflorescence apices (Fig. 2b, e). Additionally, we found *PARP2* to be expressed in heart and torpedo stage embryos (Fig. 2k, m). Hybridization with the *PARP3* probe on the same tissue led to very weak to no staining in both the shoot and the root apical meristem as well as to a weaker staining in young vascular tissue of U-stage embryos (Fig. 2r). For each transcript hybridization with a control probe in the sense orientation gave no signal (Fig. 2s–u).

**Fig. 3:**
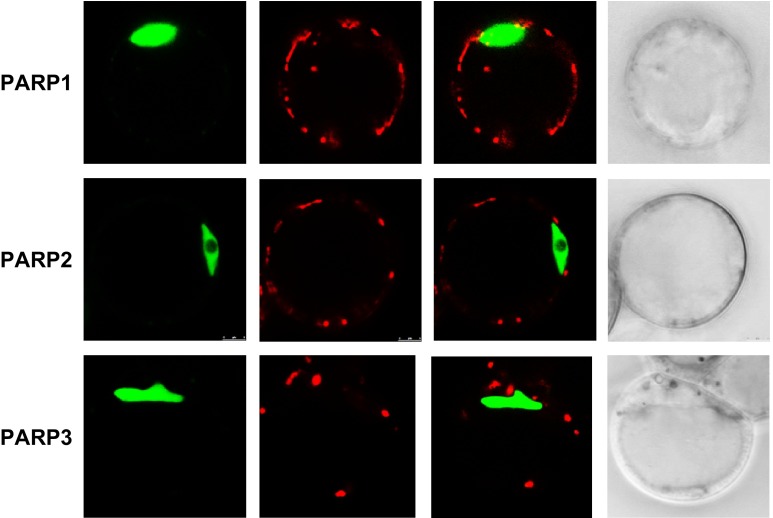
Expression of PARP1, PARP2 and PARP3 in Arabidopsis protoplast. p35S::PARP::GFP constructs were transiently expressed in protoplast derived from 3-week-old Col-0 rosette leaves grown in long day (16 h photoperiod) to detect the subcellular localization of the PARPs. With confocal microscope GFP is visualized in *green*, *red* indicates chlorophyll auto-fluorescence in chloroplasts

Based on the sequence information informatic approaches (BAR, bar.utoronto.ca, Toufighi et al. 2005), predict PARP1, to be localized to nucleus only, whereas PARP2 predicted to be present in the nucleus, mitochondria and chloroplasts and PARP3 could be highly expressed in the nucleus and slightly also in the cytoplasm. Given that such predictions have been demonstrated to be subject to error we cloned the full-length cDNAs of all three *PARP* genes and expressed them in-frame with a C-terminal GFP encoding gene under the control of the cauliflower mosaic virus (CaMV) 35S promoter (p35S). These constructs were subsequently transformed into Arabidopsis and fluorescence patterns were compared, by confocal microscopy, to the chloroplast auto-fluorescence, a nuclear control and the mitochondrial marker mitotracker within the given cell. When expressed in cell suspension culture a nuclear location of all three *PARP* gene products like that recently demonstrated by Song et al. (2015). However, protoplasts transformed with p35S::PARP1::GFP exhibited fluorescence, which was not restricted only to the nucleus, but also visible in the chloroplasts (Supplementary Fig. 1). Inspection of plants transformed with p35S::PARP2::GFP displayed strong fluorescence signals in both chloroplasts (Fig. 3D1–D3) and mitochondria ((Supplementary Fig. 1). In accordance to recent results described by (Rissel et al. 2014); PARP3 was clearly localised at high abundance in the nuclei (Supplementary Fig. 1. Additionally, we observed a partial localization of PARP3 in cytosol (Supplementary Fig. 1. Thus, whereas animal PARPs have all been identified as nuclear proteins (Ame et al. 2004; Andrabi et al. 2006), the subcellular localization of PARPs homologues in Arabidopsis leaf protoplast is not exclusively nuclear and varies slightly between the isoforms (Supplementary Fig. 1).

### Isolation (and complementation) of knockdown mutants of PARP1, PARP2 and PARP3

**Fig. 4:**
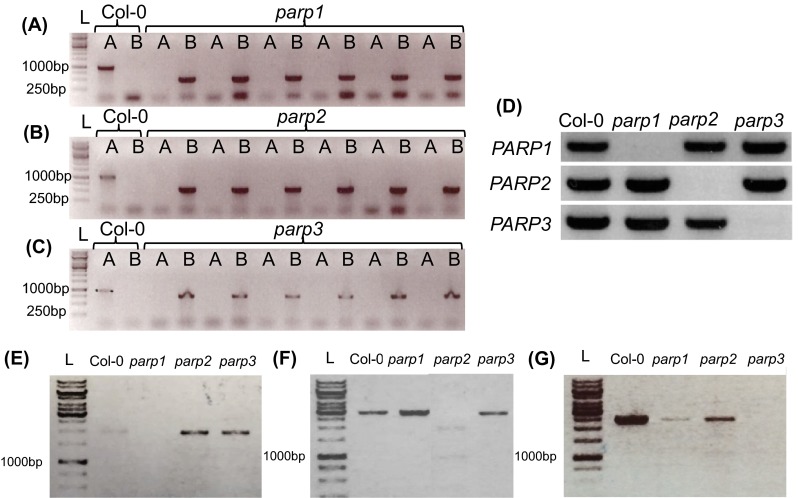
Agarose gel electrophoresis separation of genomic PCR performed in (**a**) *parp1*, (**b**) *parp2* and (**c**) *parp3* T-DNA insertion lines. PCR product with gene specific forward/reverse primers (A), PCR product with right border gene specific primer in combination with T-DNA insertion specific primer (B), 1kb gene ladder (L) was used to determine the size of PCR product (**d**). Genomic PCR was performed on *parp1*, *parp2*, *parp3* and wild type Col-0 with primer sets amplifying the genes *PARP1*, *PARP2*, *PARP3*. (**e**–**g**) Agarose gel electrophoresis of amplified *PARP* full length cDNAs in Col-0 and *parp* mutants. Leaves of 3-week-old Col-0 and *parp* lines grown in long day (16 h photoperiod) were harvested for RNA isolation and cDNA synthesis. Full length cDNA PCR was performed to determine the expression level of the following genes: (**e**) *PARP1*, (**f**) *PARP2* and (**g**) *PARP3* in wild type Col-0, *parp1*, *parp2* and *parp3*

**Fig. 5:**
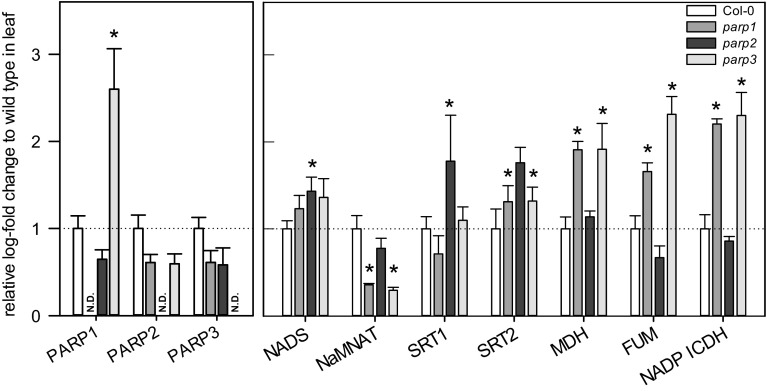
NAD^+^ hydrolase enzymatic activity via GC–MS analysis of nicotinamide (NaM) content in wild type Col-0 and the *parp* lines. Leaf whole cells as well as nuclei of plants grown in long day were isolated and extracts were desalted. Enzymatic assay was performed with addition of 1 mM NAD^+^ as substrate (without substrate in blank assays) and 60 min incubation time. Relative NaM content was normalized by the amount of NaM in blank assay, ribitol, fresh weight and incubation time. Values are mean ± SE of five to six biological replicates. *Asterisks* represent values determined by Student’s *t* test to be significantly different (*p* < 0.05) from Col-0. *N.D.* indicates not determined

The finding that the various *PARP* isoforms have distinctive expression patterns indicates that they may have functionally divergent roles depending on the tissue and developmental stage, we next isolated knockdown mutants for *PARP1, PARP2* and *PARP3*. For this purpose independent *Arabidopsis* lines that contained T-DNA elements inserted into the respective *PARP* genes were ordered from Nottingham Arabidopsis Stock Centre (NASC, Loughborough-UK). Homozygous T-DNA insertion lines for each *PARP* were confirmed by genomic PCR and assigned as *parp1*, *parp2*, and *parp3* (Fig. 4a–d). RT-PCR was next carried out to check for expression of the full-length *parp1*, *parp2*, and *parp3* transcripts (Fig. 4e–g). These studies revealed that the insertional mutants were knockouts for the allele targeted. Following this, the level of transcript of the other *PARP* genes as well as the expression of *NADS*, *NaMNAT*, *SRT1* and *SRT2* of the NAD^+^-biosynthesis pathway and the expression of mitochondrial malate dehydrogenase (*MDH*), fumarase (*FUM*) and NADP-dependent isocitrate dehydrogenase (*NADP ICDH*) in leaf tissue were investigated in each of the mutants (Fig. 5). The *PARP1*, *PARP2* and *PARP3* transcripts were non-determinant in *parp1, parp2* and *parp3* mutants, respectively (Fig. 5). The *parp1* mutant was characterized by a strong decreased expression of *NaMNAT* and between two- and threefold increased expression of *MDH*, *FUM* and *NADP ICDH*. The *parp2* mutant was additionally characterized by elevated expression of *NADS* and *SRT1* and *SRT2* but no notable difference in the expression of the examined TCA cycle transcripts. The *parp3* mutant displayed a minor decrease in the expression of *PARP*2, but a compensatory increase in the expression of *PARP1* by almost threefold (Fig. 4). Similar to the other two T-DNA lines, *parp3* showed an increased expression of *NADS*, though only *parp2* was characterized as significant. Like *parp1* line, *parp3* displayed between two and threefold increased expression of *MDH*, *FUM* and *NADP ICDH* (Fig. 5). Each of the knockout mutants was complemented by the expression of the targeted gene under the control of the 35S promoter.

### Development of a method to assay the total cellular and nuclear NAD hydrolase activities

**Fig. 6:**
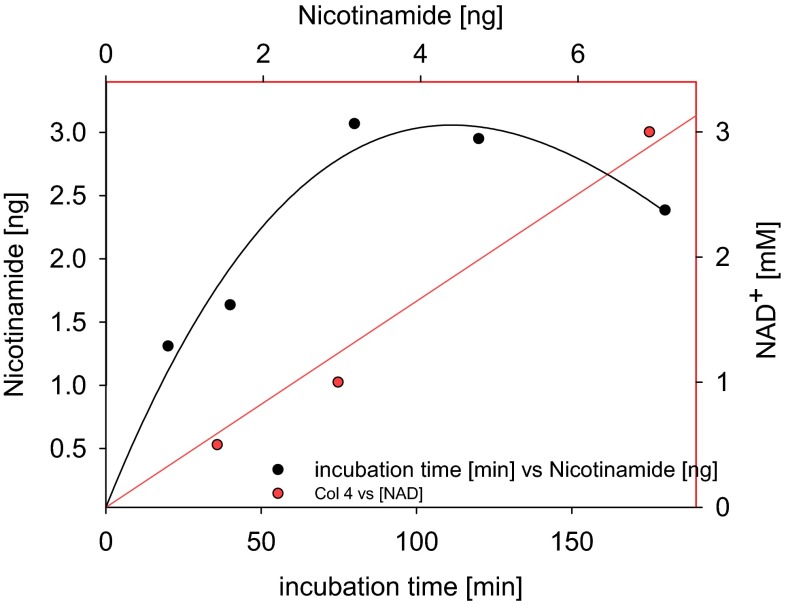
PARP enzymatic assay calibration curve. Leaf nuclei of Col-0 grown in long day (16 h photoperiod) were isolated. *Filled black circles* represent enzymatic assay performed with addition of 1 mM NAD^+^ as substrate with different incubation time (20-40-80-120-180 min). *Filled red circles* represent data of enzymatic assay performed with addition of different substrate concentration (0.5-1-3 mM NAD^+^) and 120 min incubation time

**Fig. 7:**
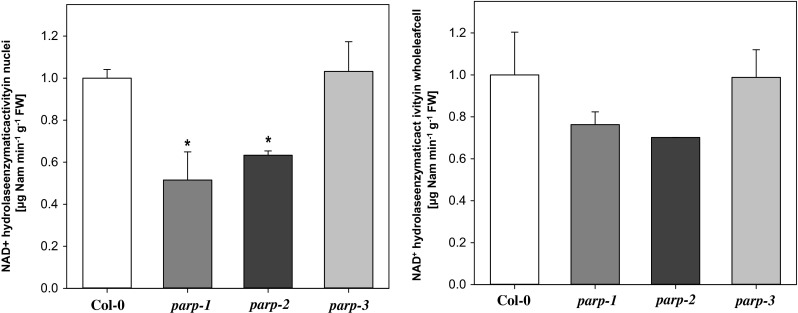
NAD^+^ hydrolase enzymatic activity via GC–MS analysis of nicotinamide (NaM) content in wild type Col-0 and the *parp* lines. Leaf whole cells as well as nuclei of plants grown in long day were isolated and extracts were desalted. Enzymatic assay was performed with addition of 1 mM NAD^+^ as substrate (without substrate in blank assays) and 60 min incubation time. Relative NaM content was normalized by the amount of NaM in blank assay, ribitol, fresh weight and incubation time. Values are mean ± SE of five to six biological replicates. *Asterisks* represent values determined by Student’s *t* test to be significantly different (*p* < 0.05) from Col-0

As mentioned above, PARP uses NAD^+^ as substrate in the salvage pathway to produce NaM via poly(ADP-ribosyl)ation. In order to investigate whether the decreases in PARP transcript resulted in similar changes at the enzyme activity level, an assay was established to determine the production of NaM in whole cell extracts as well as nuclei preparations from leaf tissue. This approach was taken given the difficulty in detecting poly(ADP-ribose) and as a means to discriminate PARP activity from that of the parallel reaction catalyzed by sirtuin (König et al. 2014) and the similar reaction catalyzed by nudix enyzmes (Hashida et al. 2009), although it is important to note that it may not discriminate PARP from other potential NAD^+^ hydrolase activities. Thus we are only able to quantify the total NAD^+^ hydrolase activity by this approach. For this purpose fresh Arabidopsis rosette leaf tissue was ground and incubated in a simple isolation buffer as described previously by (Folta and Kaufman 2006) prior to filtering through two layers of miracloth. To isolate nuclei, the filtered suspension was then lysed and centrifuged to enrich for nuclei. To remove metabolites or peptides, which could interfere with the reaction, a desalting step was carried out. The enzymatic assay was then performed with a 1 mM NAD^+^-containing enzymatic reaction buffer for 60 min at room temperature and stopped by addition of ice-cold trichloroacetic acid. The NaM produced in the end-point assay was subsequently extracted and measured by gas chromatography-mass spectrometry (GC–MS) as detailed in the “Materials and methods” section. Given that this was an assay novel to us, before analyzing the mutant lines we verified that it was linear with respect to both time and protein concentration (data not shown). The cellular NAD^+^ hydrolysis activity of wild type Arabidopsis observed (Fig. 6) was similar to those previously reported for PARP in mammals and maize (Grube and Bürkle 1992; Tian et al. 1999). A reduced activity was observed for the *parp1* and *parp*2 mutants in both extracts of whole leaf extracts and nuclei preparations, although the reduction was statistically significant only for the nuclei (Fig. 7). Expectedly, the activity in *parp3* mutant was at wild type level, since *PARP3* is not highly expressed in vegetative tissue, however importantly the enhanced expression of PARP1 in this line did not result in an increased overall PARP activity.

### Characteristics of seed germination and root growth

**Fig. 8:**
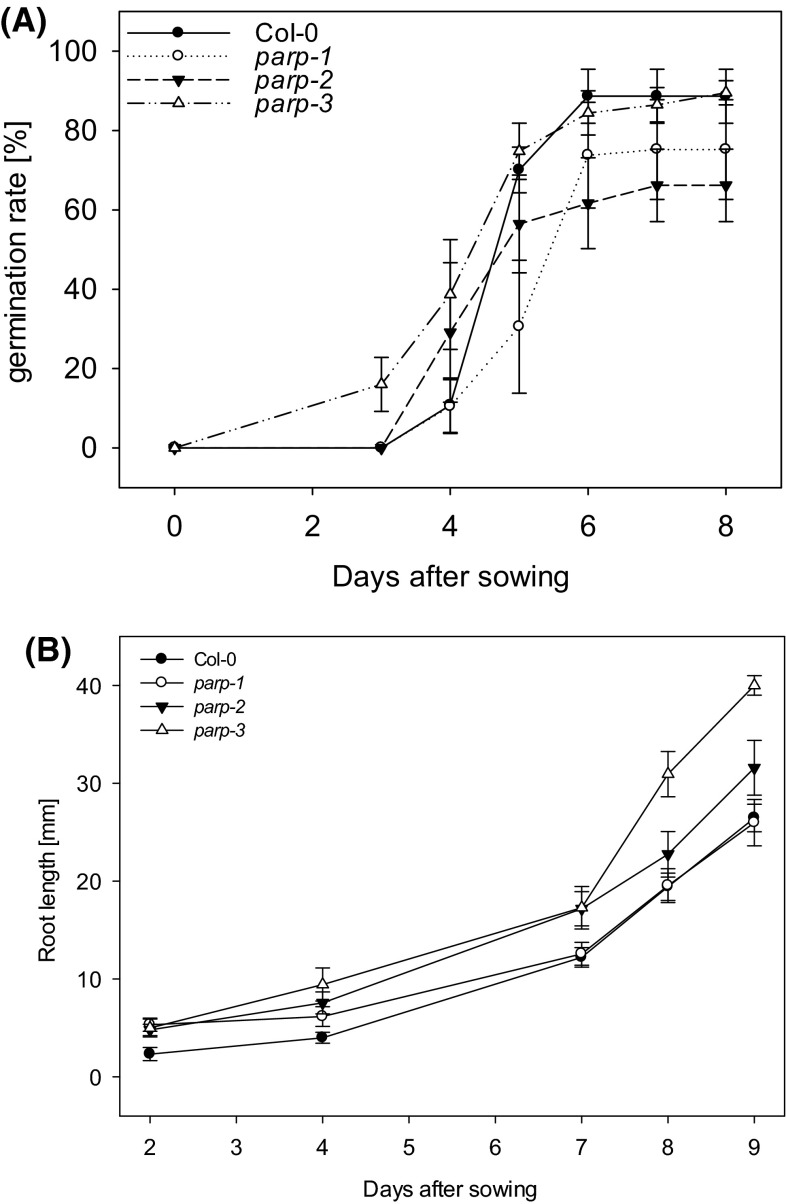
Germination rate and root elongation rate of *parp* mutant, complemented lines c*PARP* and wild type Col-0. Sterilized seeds were grown on half-strength Murashige and Skoog (MS) agar supplemented with 1 % sucrose under long day condition (16 h photoperiod). **a** Germination rate is expressed as total germination in percentage ±SE of three repetitions and **b** root length is expressed as mean ± SE. *Asterisks* represent values determined by Student’s *t* test to be significantly different (*p* < 0.05 and *p* < 0.01) from Col-0. *n.g.* no germination

To study the impact of *PARP* inactivation on plant growth we next compared seed germination of the mutants with that of the wild type in the presence of sucrose. Using freshly harvested seeds we observed that on half-strength Murashige and Skoog (MS) agar with 1 % sucrose *parp3* started to germinate after 3 days, thus earlier than Col-0, *parp1* and *parp2* mutants (Fig. 8a). While after 4 days *parp2* and *parp3* lines continued to show a higher germination rate compared to wild type, the final germination rate of *parp3* was similar to Col-0, whereas the other two insertion lines revealed an overall slower and slightly reduced germination rate (Fig. 8a). A similar germination rate was observed on half-strength MS agar without 1 % sucrose supplement, indicating, that *PARP3* deficient plants were capable of sugar independent germination. More importantly by using complemented mutant lines we could observe the rescue of the wild type phenotype (Fig. 8a).

As a next experiment the root development of homozygous lines was determined on 2MS medium (Fig. 8b). A higher root elongation rate was generally observed in all mutant lines in comparison to wild type. Although *parp1* was only significantly higher than Col-0 after 4 days, *parp2* and *parp3* were significantly higher than Col-0 throughout the experiment (Fig. 8b). Complemented mutant lines c*PARP*s, however, displayed a reduced root elongation compared to their respective mutants. Notably this result is somewhat different between the lines indicating functionally non-redundant roles for the isoforms.

### Metabolic alterations in leaves of parp mutants

**Fig. 9:**
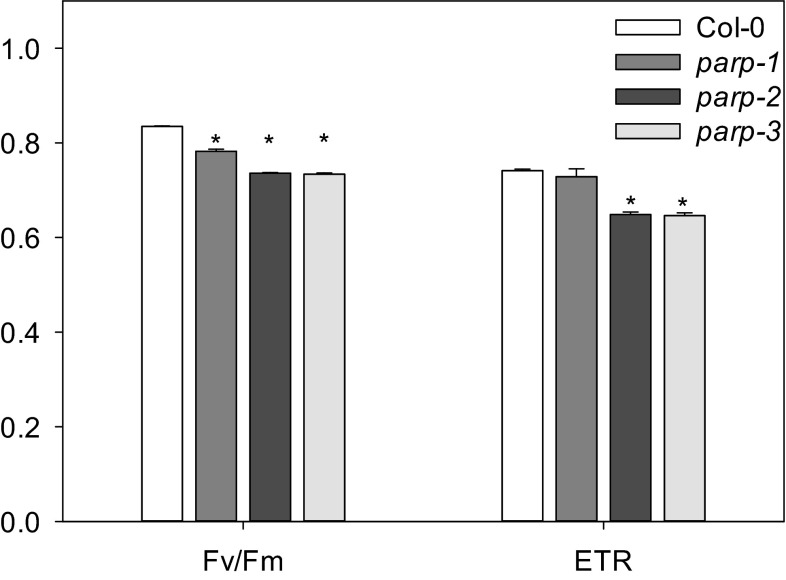
Measurement of chlorophyll fluorescence and the efficiency of electron transport in dark adapted leaves of *parp* lines in comparison to wild type Col-0. Leaves of 4-week-old plants grown under long day (16 h photoperiod) were clamped in darkness for 10 min prior applying a light source to determine the photochemical efficiency of photosystem II [ratio of maximal variable fluorescence to maximum yield of fluorescence (F_v_/F_m_)] and the electron transport rate (ETR). Values are mean ± SE of seven biological replicates and *asterisks* represent values determined by Student’s *t* test to be significantly different (*p* < 0.05) from Col-0

**Fig. 10:**
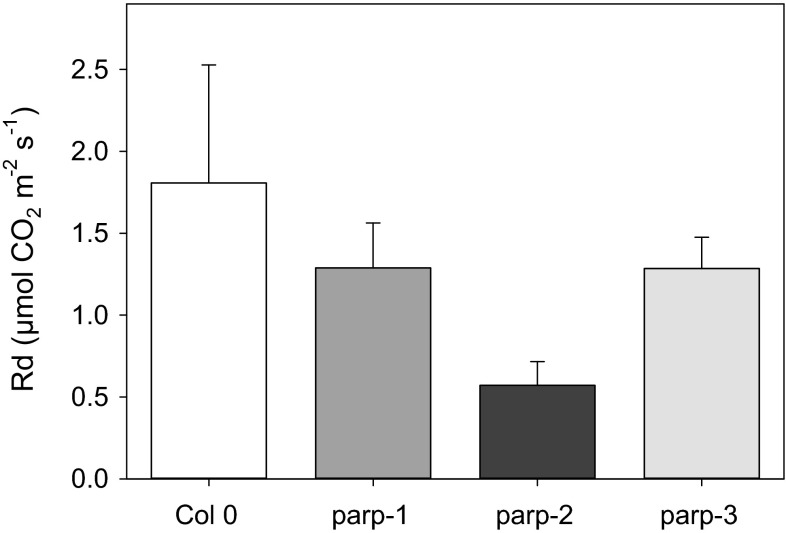
Effect of decreased PARP activity on photosynthetic parameters. 4-Weeks-old in long day (16 h photoperiod) grown *parp* lines and wild type were dark adapted 30 min prior exposure to light sources of various intensities. Stomatal conductance, assimilation rate and stomatal transpiration rate were determined. Values are mean ± SE of 4–5 biological replicates and *asterisks* represent values determined by student’s *t* test to be significantly different (*p* < 0.05) from Col-0

**Fig. 11:**
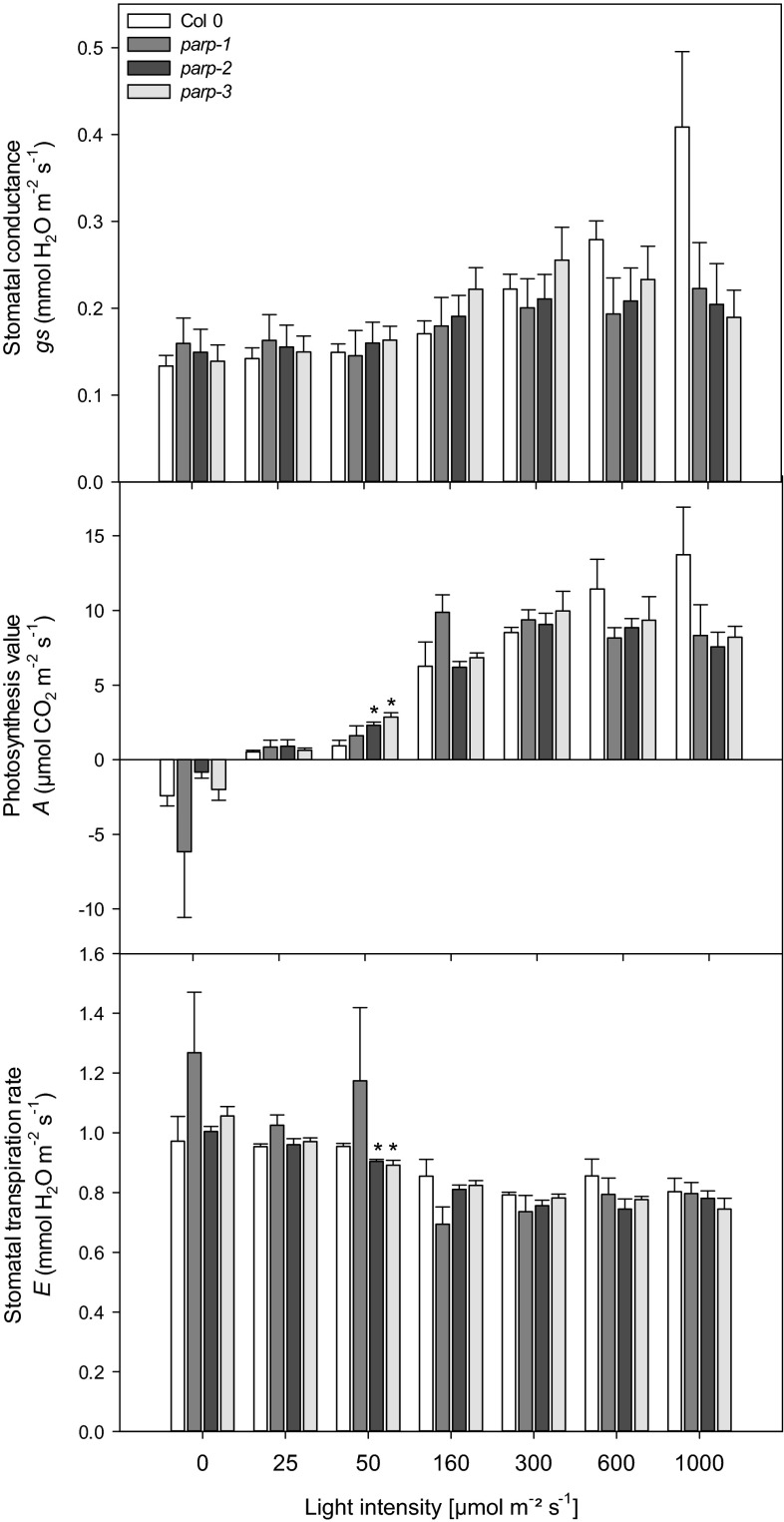
Effect of decreased PARP activity on respiration of 4-week-old plants. *parp* lines and wild type plants grown in long day (16 h photoperiod) were dark adapted 30 min prior exposure to light source. Values are mean ± SE of 4–5 biological replicates and *asterisks* represent values determined by Student’s *t* test to be significantly different (*p* < 0.05) from Col-0

To further characterize the roles of the independent PARP isoforms we next performed metabolite profiling in leaves of the mutants. Despite lacking changes in total chlorophyll content, the chlorophyll a/b ratio was reduced in *parp*2 and increased in *parp1*, whereas no differences between wild type and *parp3* were detected. We further investigated whether these changes in chlorophyll might be associated with photosynthetic activity observing minor yet significant reductions in the PSII maximum efficiency after dark adaptation (*F*_v_/*F*_m_) as well as in electron transport rate (ETR; not significant in *parp1*) compared to Col-0 (Fig. 9). It is worth mentioning that the changes in *F*_v_/*F*_m_ and ETR were minor in plants of all genotypes, typically indicative of a lack of major stress and rather associated with general changes in metabolism (Essemine et al. 2012). Additionally, gas exchange was measured directly in 4-week-old plants growing under long day condition, under photon flux densities (PFDs) that ranged from 25 to 1000 μmol m^−2^ s^−1^. All *parp* mutants exhibited unaltered assimilation rates, stomatal conductance, intracellular and ambient CO_2_ ration (*C*_i_/*C*_a_) and transpiration rates (Fig. 10). Furthermore, we measured the rate of dark respiration using via infrared gas-exchange analyses (Fig. 11). These measurements revealed that in all *parp* mutants there is a tendency of reduction in dark respiration, and although this was not significant in any of the genotypes, the alteration in *parp2* was higher than in *parp1* and *parp3* (Fig. 11). When taking together, alongside the results of in *F*_v_/*F*_m_ and ETR, these data strongly suggest that photosynthetic machinery is not compromised in the absence of any individual *parp* isozymes.

To further elucidate the role of *PARP*s in *Arabidopsis thaliana* we next used an established GC–MS platform that affords good coverage of the major metabolites of primary metabolism (Fernie et al. 2004; Lisec et al. 2006) to quantify the relative metabolite levels in leaf samples of *parp* mutants in order to confirm the suggested alteration in plant metabolism. These studies revealed considerable changes in the levels of a wide range of organic acids, amino acids and sugars (Table 2). In order to characterize changes in the metabolome Tukeys tests were employed wherein the wild type was independently compared to each mutant and it´s respective complemented line we describe a change as significant only when it is altered in the mutant and not in the complemented line or when it is altered in the mutant and the complemented line is significantly different from the mutant and reverting its change. The levels of many amino acids were reduced significantly in one or more of the mutant lines for example alanine and GABA in *parp1*, isoleucine and ornithine in *parp2* and *parp3* and glycine, phenylalanine and tyrosine in *parp2* alone. By contrast, aspartate levels were increased in *parp1* and *parp3* and glutamine in *parp2 and parp3.*

**Table 2:** Metabolite analysis in leaves of 3-week-old Col-0 and *parp* mutants grown in long day (16 h photoperiod)

Amino acids	Col-0	*parp1*	*cPARP1*	Turkey	*parp2*	*cPARP2*	Turkey	*parp3*	*cPARP3*	Turkey
	AVG ± SE	AVG ± SE	AVG ± SE		AVG ± SE	AVG ± SE		AVG ± SE	AVG ± SE	
Alanine	1.000 ± 0.029	0.745 ± 0.073	1.266 ± 0.108	a/b/c	1.064b ± 0.124	1.155 ± 0.153	a/ab/b	1.080 ± 0.025	0.998 ± 0.067	a/ab/b
Arginine	1.000 ± 0.133	1.442 ± 0.280	1.338 ± 0.072	a/b/b	0.745 ± 0.186	1.405 ± 0.074	a/a/b	1.369 ± 0.082	1.832 ± 0.099	a/a/b
Aspartate	1.000 ± 0.058	1.520 ± 0.103	1.057 ± 0.044	a/b/ab	1.048 ± 0.139	1.070 ± 0.036	a/a/a	1.313 ± 0.069	1.050 ± 0.096	a/b/ab
GABA	1.000 ± 0.092	0.759 ± 0.105	1.182 ± 0.050	a/b/ab	0.892 ± 0.095	1.127 ± 0.059	a/a/a	0.886 ± 0.114	0.894 ± 0.094	a/a/a
Glutamate	1.000 ± 0.055	1.123 ± 0.046	1.308 ± 0.069	a/ab/b	1.081 ± 0.113	1.202 ± 0.132	a/a/a	1.296 ± 0.084	1.393 ± 0.089	a/b/b
Glutamine	1.000 ± 0.061	1.587 ± 0.121	1.305 ± 0.075	a/b/b	1.733 ± 0.369	0.890 ± 0.126	a/b/ab	1.940 ± 0.132	0.915 ± 0.119	a/b/ab
Glycine	1.000 ± 0.027	0.970 ± 0.193	1.803 ± 0.160	a/a/b	0.573 ± 0.098	2.123 ± 0.117	a/b/c	0.743 ± 0.105	1.707 ± 0.233	a/b/c
Isoleucine	1.000 ± 0.028	0.832 ± 0.097	0.954 ± 0.047	a/b/ab	0.670 ± 0.041	0.981 ± 0.034	a/b/ab	0.843 ± 0.053	0.885 ± 0.086	a/b/ab
Methionine	1.000 ± 0.039	1.281 ± 0.139	1.472 ± 0.052	a/b/b	1.066 ± 0.129	1.323 ± 0.070	a/a/b	1.452 ± 0.119	1.680 ± 0.187	a/b/b
Ornithine	1.000 ± 0.029	0.897 ± 0.236	1.642 ± 0.087	a/a/b	0.467 ± 0.105	1.128 ± 0.068	a/b/ab	0.521 ± 0.103	0.920 ± 0.143	a/b/ab
Phenylalanine	1.000 ± 0.024	1.020 ± 0.086	1.029 ± 0.045	a/a/a	0.856 ± 0.013	0.955 ± 0.008	a/b/ab	1.015 ± 0.072	0.897 ± 0.049	a/a/a
Proline	1.000 ± 0.033	0.981 ± 0.093	2.492 ± 0.186	a/a/b	0.893 ± 0.101	2.707 ± 0.188	a/a/b	1.295 ± 0.114	3.300 ± 0.274	a/b/c
Tyrosine	1.000 ± 0.042	0.859 ± 0.195	1.288 ± 0.133	ab/a/b	0.585 ± 0.115	0.967 ± 0.052	a/b/ab	0.611 ± 0.076	0.587 ± 0.106	a/b/b
*Acids and hydroxy acids*										
Dehydroascorbate	1.000 ± 0.058	1.488 ± 0.164	1.145 ± 0.075	a/b/ab	1.449 ± 0.109	1.409 ± 0.077	a/b/b	2.262 ± 0.248	1.861 ± 0.100	a/b/b
Fumarate	1.000 ± 0.088	1.524 ± 0.110	1.807 ± 0.104	a/b/b	0.917 ± 0.210	1.379 ± 0.182	a/ab/b	1.295 ± 0.126	1.759 ± 0.110	a/b/c
Glycerate	1.000 ± 0.060	0.782 ± 0.053	0.903 ± 0.047	a/b/ab	1.071 ± 0.158	1.051 ± 0.027	a/a/a	1.255 ± 0.140	0.873 ± 0.094	a/b/ab
Lactate dimer	1.000 ± 0.035	0.805 ± 0.052	0.832 ± 0.083	a/b/ab	0.702 ± 0.005	1.019 ± 0.029	a/b/ab	0.723 ± 0.042	0.539 ± 0.033	a/b/c
Malate	1.000 ± 0.090	1.652 ± 0.144	1.135 ± 0.057	a/b/ab	1.056 ± 0.122	1.139 ± 0.071	a/a/a	1.462 ± 0.106	1.517 ± 0.038	a/b/b
Pyroglutamate	1.000 ± 0.043	1.440 ± 0.079	1.138 ± 0.044	a/b/c	1.266 ± 0.107	1.058 ± 0.031	a/b/ab	1.622 ± 0.109	1.064 ± 0.133	a/b/ab
Succinate	1.000 ± 0.048	1.260 ± 0.049	1.306 ± 0.063	a/b/b	1.023 ± 0.047	1.419 ± 0.063	a/a/b	1.176 ± 0.080	1.174 ± 0.095	a/b/ab
Threonate	1.000 ± 0.065	1.147 ± 0.066	1.090 ± 0.037	a/b/ab	0.728 ± 0.050	0.940 ± 0.044	a/b/ab	0.960 ± 0.080	1.040 ± 0.064	a/a/a
*Sugars*										
Fructose	1.000 ± 0.106	1.424 ± 0.519	0.962 ± 0.035	a/a/a	0.637 ± 0.026	0.782 ± 0.045	a/b/ab	1.004 ± 0.102	1.154 ± 0.114	a/a/a
Glucose	1.000 ± 0.039	1.226 ± 0.108	1.117 ± 0.071	a/b/ab	0.912 ± 0.085	1.108 ± 0.046	a/a/a	1.294 ± 0.130	0.913 ± 0.098	a/b/ab
Raffinose	1.000 ± 0.096	1.363 ± 0.488	1.554 ± 0.113	a/a/a	0.637 ± 0.072	0.992 ± 0.106	a/b/ab	1.684 ± 0.814	1.771 ± 0.205	a/a/a
Ribose	1.000 ± 0.018	1.009 ± 0.011	1.108 ± 0.043	a/a/b	1.047 ± 0.016	0.935 ± 0.040	ab/a/b	1.008 ± 0.009	1.050 ± 0.055	a/a/a
Sucrose	1.000 ± 0.032	1.129 ± 0.039	1.008 ± 0.022	a/b/ab	1.018 ± 0.043	1.097 ± 0.022	a/a/a	1.507 ± 0.039	1.150 ± 0.028	a/b/ab
Trehalose	1.000 ± 0.098	1.107 ± 0.197	1.178 ± 0.050	a/a/a	0.625 ± 0.023	0.943 ± 0.057	a/b/ab	1.781 ± 0.453	1.627 ± 0.097	a/b/ab
*Sugar alcohols*										
Galactinol	1.000 ± 0.082	1.578 ± 0.798	1.191 ± 0.030	a/a/a	0.502 ± 0.144	1.027 ± 0.052	a/b/ab	0.780 ± 0.141	0.949 ± 0.050	a/a/a
Glycerol	1.000 ± 0.027	0.775 ± 0.084	1.030 ± 0.040	a/b/ab	0.685 ± 0.018	1.156 ± 0.048	a/b/c	0.637 ± 0.043	0.951 ± 0.087	a/b/ab
myo-Inositol	1.000 ± 0.054	1.171 ± 0.107	1.022 ± 0.038	a/b/ab	0.875 ± 0.035	0.859 ± 0.064	a/a/a	1.293 ± 0.047	1.301 ± 0.091	a/b/b
Sorbitol	1.000 ± 0.050	1.411 ± 0.350	1.162 ± 0.090	a/b/ab	0.526 ± 0.028	1.089 ± 0.034	a/b/ab	0.504 ± 0.080	1.318 ± 0.158	a/b/c
*Other metabolites*										
Putrescine	1.000 ± 0.099	1.059 ± 0.322	1.063 ± 0.050	a/b/ab	0.878 ± 0.132	0.889 ± 0.046	a/a/a	1.323 ± 0.189	0.953 ± 0.078	a/b/ab
Spermidine	1.000 ± 0.075	0.844 ± 0.054	1.271 ± 0.054	a/a/b	0.955 ± 0.183	1.020 ± 0.066	a/a/a	0.869 ± 0.064	1.026 ± 0.169	a/a/a

The levels of malate and dehydroascorbate were increased in *parp*-*1* whilst succinate was increased in *parp*-*1.* By contrast, glycerate was decreased in *parp*-*1* and *parp*-*3*, lactate was decreased in *parp*-*1* and *parp*-*2* and pyroglutamate was increased in all lines. Threonate increased in *parp*-*1* but decreased in *parp*-*2.* Furthermore, an increase in Glu and Suc content in *parp*-*1* and *parp*-*3*, although not in was observed. Conversely the levels of fructose, raffinose and trehalose were decreased in *parp*-*2*, whilst levels of the latter increased in *parp*-*3* and minor alterations in sugar alcohols and polyamines were also apparent.

**Fig. 12:**
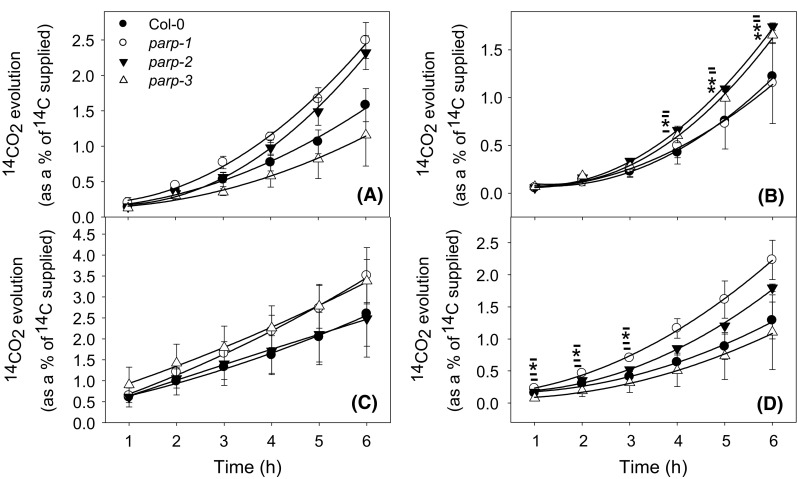
Evolution of ^14^CO_2_ in wild type Col-0 and *parp* mutants grown in long day (16 h photoperiod). Leaf discs of 4-week-old plants were isolated and incubated in MES-KOH solution (pH 6.5) supplemented with 2.32 kBq mL^−1^ of **a** D-[1-^14^C]-, **b** D-[2-^14^C], **c** D-[3,4-^14^C]- or **d** D-[6-^14^C]-glucose. The ^14^CO_2_ release was captured hourly and quantified by liquid scintillation counting. Values are mean ± SE of three biological replicates each d-glucose labelling. *Asterisk* represent values determined by Student’s *t* test to be significantly different (*p* < 0.05) from Col-0

We next directly evaluated the rate of light respiration in the mutant lines. For this purpose we recorded the evolution of ^14^CO_2_ following the incubation of leaf discs in positional-labeled ^14^C-glucose molecules in order to assess the relative rate of flux through the oxidative pentose phosphate pathway (OPPP) and the TCA cycle (Fig. 12). For this leaf discs were incubated in the light and supplied with [1-^14^C]-Glc, [2-^14^C]-Glc, [3,4-^14^C]-Glc, or [6-^14^C]-Glc over a period of 6 h. During this time the ^14^CO_2_ evolved was collected at hourly intervals. Carbon dioxide can be released from the C1 position, and to a lesser extent the C2 position, by the action of enzymes that are not associated with mitochondrial respiration. On the other hand carbon dioxide released from the C3,4 positions of glucose are directly associated with the activity of enzymes connected to the mitochondrial respiration (Nunes-Nesi et al. 2007). Thus, the ratio of carbon dioxide released from C3,4 to C1 positions of glucose provides a strong indication of the relative rate of the TCA cycle in regard to other processes of carbohydrate oxidation. An interesting pattern was observed when the relative ^14^CO_2_ release of the mutant and wild type lines is compared for the various fed substrates regardless of the labeled position in the substrate. For instance following either [1-^14^C]-(Fig. 12a) or [2-^14^C]-labeling (Fig. 12b), CO_2_ release of *parp1* was higher than wild type, while for *parp2* plants a higher CO_2_ release for [1-^14^C] and a similar CO_2_ release for [2-^14^C] was observed. In addition *parp3* plants showed a reduced CO_2_ release for [1-^14^C] and an increased CO_2_ release for [2-^14^C]. The CO_2_ release from the position [3,4-^14^C] also revealed an interesting pattern (Fig. 12c) with both *parp1* and *parp3* displayed increased evolution compared to wild type, while *parp2* was invariant from wild type. Additionally, the CO_2_ release from the C6 position (Fig. 12d) of *parp1* and *parp2* are higher than wild type, while that from *parp3* was similar to wild type. There was, furthermore, a shift in the ratio of CO_2_ evolution from the various labelled substrates, with the relative release from the C3,4 positions in relation to C1 in comparison to wild type plants. Ratios were as follows: wild type = 0.54 ± 0.07; *parp1* = 0.37 ± 0.04; *parp2* = 0.61 ± 0.06, and *parp3* = 0.94 ± 0.15). These data suggested a tendency of a higher proportion of carbohydrate oxidation performed by the TCA cycle in *parp2* and *parp3* plants (significant in *parp3*).

**Fig. 13:**
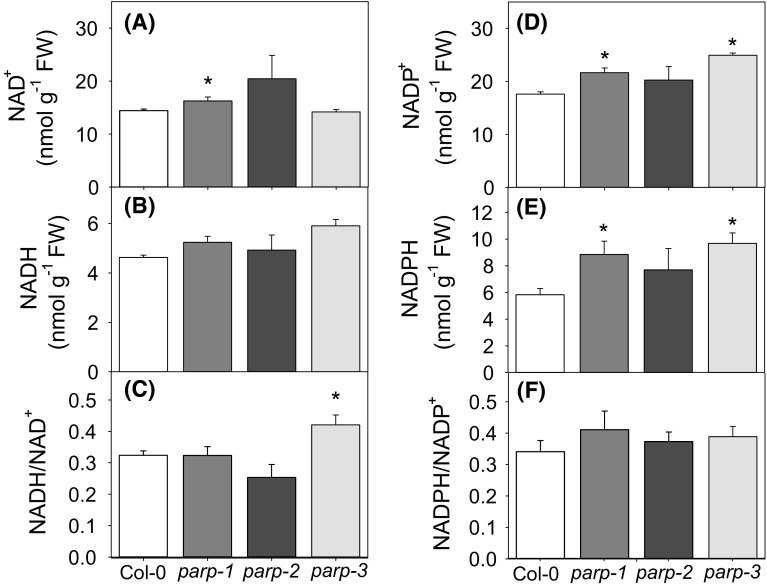
Pyridine nucleotide levels and ratios in leaves of WT Col-0 and *parp* lines. Leaf materials of 3-week-old plants grown in long day (16 h photoperiod) were harvested in the *middle* of the light period and extracted to determine the redox status of the *parp* lines in comparison to wild type. Detected content of NAD(H) and NADP(H) are given in nmol g^−1^ FW with NAD^+^ content (**a**), NADH content (**b**), NADH/NAD^+^ ratio (**c**), NADP^+^ content (**d**), NADPH content (**e**) and NADPH/NADP^+^ ratio (**f**). Values are mean ± SE of three to eight biological replicates. *Asterisk* represent values determined by the Student’s *t* test to be significantly different (*p* < 0.05) from wild type

Since the poly(ADP-ribosyl)ation activity might also be associated with the consumption of oxidized coenzyme NAD^+^, it is reasonable to anticipate that reduction in the activity of PARP enzymes might affects the redox balance in the knockout plants. Therefore, we decided to determine the levels of pyridine dinucleotides in the leaves of wild type and mutant plants (Fig. 13). An increase in the level of NAD^+^ was observed in *parp1)* while no significant differences were observed for *parp2* and *parp*-*3* plants (Fig. 13a). Although a tendency of increased NADH content was observed for all lines, no significant changes were detected in the content of this dinucleotide (Fig. 13b). In addition only in *parp3* was a decreased NADH/NAD^+^ ratio observed (Fig. 13c). Significant increases in the content of NADP^+^ and NADPH were, however, observed for *parp1* and *parp3* plants (Fig. 13d, e). Despite these changes there were no significant alterations in the NADPH/NADP^+^ ratio (Fig. 13f).

### Metabolic profile in roots of parp mutants

To follow the repertoire of metabolic changes that might explain the reasons behind the root phenotype in *parp* mutants we have also performed an extensive metabolite profiling in roots of 4-week-old Arabidopsis plants grown under long day conditions (Table 3). Changes were statistically assessed in the same manner as above for the shoots there were no changes in protein, nitrate or total amino acid levels nor in sucrose or fructose but the levels of glucose were reduced in *parp*-*1* and *parp*-*2* whilst the levels of starch were decreased in *parp*-*1* and *parp*-*3.* Perhaps surprisingly, there were very few changes in the levels of metabolites determined by GC–MS in either *parp*-*1* or *parp*-*2.* The *parp*-*1* mutant displayed increases in asparagine and beta-alanine, whilst *parp*-*2* was unaltered whilst *parp*-*3* displayed the decrease in glucose we observed by spectrophotometric methods. By contrast, *parp*-*3* displayed decreased levels of intermediates of the photorespiratory pathway namely glycine and serine as well as glycerate and glycolate as well as the stress amino acids GABA and proline and the TCA cycle intermediates malate and succinate. Conversely, the levels of methionine and phenylalanine as well as those of phosphorate were increased in *parp*-*3.* In addition, the levels of raffinose, xylose, galactionol, myo-inositol, threitol and urea were all decreased in *parp*-*3.*

**Table 3:** Metabolite analysis in leaves of 3-week-old Col-0 and *parp* mutants grown in long day (16 h photoperiod)

AminoAcids	Col-0	*parp1*	*cPARP1*	Turkey	*parp2*	*cPARP2*	Turkey	*parp3*	*cPARP3*	Turkey
	AVG ± SE	AVG ± SE	AVG ± SE		AVG ± SE	AVG ± SE		AVG ± SE	AVG ± SE	
Alanine	1.000 ± 0.153	0.817 ± 0.304	0.828 ± 0.691	a/a/a	0.893 ± 0.323	1.748 ± 0.490	a/a/a	0.182 ± 0.019	1.946 ± 0.213	a/b/c
Asparagine	1.000 ± 0.430	3.640 ± 0.837	1.045 ± 0.327	a/b/ab	1.965 ± 0.487	1.488 ± 0.179	a/a/a	1.522 ± 0.383	1.621 ± 0.340	a/a/a
Aspartate	1.000 ± 0.233	1.741 ± 0.178	0.867 ± 0.539	a/a/a	1.128 ± 0.268	1.083 ± 0.114	a/a/a	0.829 ± 0.085	0.965 ± 0.038	a/a/a
Beta-alanine	1.000 ± 0.243	2.846 ± 0.669	0.783 ± 0.440	a/b/ab	2.212 ± 1.061	1.484 ± 0.668	a/a/a	0.533 ± 0.031	0.515 ± 0.050	a/a/a
GABA	1.000 ± 0.066	1.378 ± 0.110	0.749 ± 0.437	a/a/a	1.033 ± 0.251	1.394 ± 0.090	a/a/a	0.504 ± 0.013	1.001 ± 0.031	a/b/ab
Glutamine	1.000 ± 0.189	0.861 ± 0.088	1.281 ± 1.200	a/a/a	1.398 ± 0.455	1.397 ± 0.329	a/a/a	0.421 ± 0.074	1.989 ± 0.399	a/a/b
Glycine	1.000 ± 0.068	1.415 ± 0.124	1.261 ± 0.881	a/a/a	1.336 ± 0.378	1.317 ± 0.201	a/a/a	0.468 ± 0.036	0.902 ± 0.061	a/b/ab
Isoleucine	1.000 ± 0.405	2.403 ± 0.610	1.102 ± 0.522	a/a/a	1.607 ± 0.696	1.341 ± 0.261	a/a/a	1.688 ± 0.207	0.522 ± 0.025	ab/a/b
Methionine	1.000 ± 0.406	3.134 ± 0.931	1.384 ± 0.599	a/a/a	1.481 ± 0.591	1.489 ± 0.466	a/a/a	2.568 ± 0.249	0.522 ± 0.017	a/b/ab
Ornithine	1.000 ± 0.451	2.252 ± 0.447	2.056 ± 1.181	a/a/a	3.327 ± 1.170	1.963 ± 0.236	a/a/a	2.521 ± 0.430	2.270 ± 0.790	a/a/a
Phenylalanine	1.000 ± 0.285	2.141 ± 0.341	1.678 ± 0.717	a/a/a	1.457 ± 0.466	1.700 ± 0.214	a/a/a	2.385 ± 0.304	0.861 ± 0.155	a/b/ab
Proline	1.000 ± 0.218	0.425 ± 0.181	0.456 ± 0.411	a/a/a	0.550 ± 0.228	0.852 ± 0.190	a/a/a	0.081 ± 0.011	1.034 ± 0.027	a/b/ab
Pyruvate	1.000 ± 0.053	0.971 ± 0.106	2.199 ± 1.463	a/a/a	0.945 ± 0.190	1.359 ± 0.172	a/a/a	0.872 ± 0.092	1.346 ± 0.062	a/a/b
Serine	1.000 ± 0.053	1.454 ± 0.102	0.819 ± 0.596	a/a/a	1.152 ± 0.284	1.565 ± 0.099	a/a/a	0.303 ± 0.038	1.307 ± 0.042	a/b/c
Threonine	1.000 ± 0.086	1.462 ± 0.092	1.048 ± 0.602	a/a/a	1.137 ± 0.269	1.253 ± 0.091	a/a/a	0.766 ± 0.075	1.157 ± 0.026	ab/a/b
Valine	1.000 ± 0.288	2.024 ± 0.390	0.998 ± 0.492	a/a/a	1.407 ± 0.498	1.263 ± 0.188	a/a/a	1.279 ± 0.101	0.719 ± 0.021	a/a/a
*Acids andhydroxy acids*										
Glycerate	1.000 ± 0.039	2.087 ± 0.338	0.987 ± 0.792	a/a/a	1.505 ± 0.529	1.440 ± 0.161	a/a/a	0.249 ± 0.057	0.987 ± 0.058	a/b/ab
Glycolate	1.000 ± 0.083	1.204 ± 0.230	1.851 ± 1.038	a/a/a	1.150 ± 0.233	1.031 ± 0.102	a/a/a	0.656 ± 0.048	0.948 ± 0.112	a/b/ab
Malate	1.000 ± 0.085	1.305 ± 0.269	0.662 ± 0.473	a/a/a	0.814 ± 0.217	1.239 ± 0.166	a/a/a	0.265 ± 0.012	0.900 ± 0.022	a/b/ab
Nicotinate	1.000 ± 0.161	1.464 ± 0.354	1.356 ± 0.560	a/a/a	0.799 ± 0.221	1.296 ± 0.353	a/a/a	1.205 ± 0.054	0.813 ± 0.095	a/a/a
Phosphorate	1.000 ± 0.591	2.936 ± 0.933	1.422 ± 0.353	a/a/a	2.015 ± 1.057	1.556 ± 0.832	a/a/a	2.510 ± 0.292	0.203 ± 0.016	a/b/ab
Succinate	1.000 ± 0.067	1.233 ± 0.133	0.731 ± 0.447	a/a/a	1.305 ± 0.403	0.931 ± 0.114	a/a/a	0.298 ± 0.014	0.674 ± 0.133	a/b/ab
*Sugars*										
Fructose	1.000 ± 0.102	0.539 ± 0.022	1.748 ± 0.655	ab/a/b	0.781 ± 0.106	0.963 ± 0.118	a/a/a	0.919 ± 0.012	1.185 ± 0.077	a/a/a
Glucose	1.000 ± 0.173	0.260 ± 0.014	1.464 ± 0.971	a/a/a	0.637 ± 0.190	0.848 ± 0.157	a/a/a	0.567 ± 0.008	1.198 ± 0.044	a/b/a
Melezitose	1.000 ± 0.161	1.028 ± 0.175	0.841 ± 0.630	a/a/a	0.793 ± 0.239	1.017 ± 0.159	a/a/a	0.410 ± 0.068	0.604 ± 0.106	a/a/a
Raffinose	1.000 ± 0.191	0.561 ± 0.221	0.879 ± 0.688	a/a/a	0.679 ± 0.229	1.560 ± 0.359	a/a/a	0.160 ± 0.035	1.429 ± 0.077	a/b/ab
Sorbose	1.000 ± 0.109	0.485 ± 0.023	1.757 ± 0.749	ab/a/b	0.833 ± 0.101	0.960 ± 0.124	a/a/a	0.891 ± 0.008	1.204 ± 0.078	ab/a/b
Sucrose	1.000 ± 0.456	2.631 ± 0.756	2.011 ± 1.446	a/a/a	1.380 ± 0.695	1.488 ± 0.762	a/a/a	1.490 ± 0.209	0.248 ± 0.007	ab/a/b
Xylose	1.000 ± 0.113	0.668 ± 0.079	0.844 ± 0.578	a/a/a	0.921 ± 0.235	1.326 ± 0.182	a/a/a	0.320 ± 0.024	1.226 ± 0.021	a/b/ab
*Sugar alcohols*										
Galactinol	1.000 ± 0.113	0.741 ± 0.307	0.919 ± 0.638	a/a/a	0.947 ± 0.300	2.582 ± 0.688	a/a/a	0.221 ± 0.048	1.428 ± 0.027	a/b/c
myo-Inositol	1.000 ± 0.077	0.678 ± 0.036	0.878 ± 0.436	a/a/a	0.802 ± 0.168	0.950 ± 0.049	a/a/a	0.468 ± 0.042	0.868 ± 0.039	a/b/ab
Threitol	1.000 ± 0.053	1.403 ± 0.183	1.358 ± 0.920	a/a/a	1.739 ± 0.706	1.056 ± 0.060	a/a/a	0.629 ± 0.022	1.279 ± 0.104	a/b/c
*Other metabolites*										
Putrescine	1.000 ± 0.115	0.869 ± 0.061	1.254 ± 0.747	a/a/a	0.989 ± 0.237	2.106 ± 0.392	a/a/b	0.844 ± 0.410	1.404 ± 0.070	a/a/a
Urea	1.000 ± 0.042	0.826 ± 0.051	1.444 ± 0.858	a/a/a	0.745 ± 0.177	1.023 ± 0.096	a/a/a	0.840 ± 0.028	1.154 ± 0.033	a/b/c
Protein	1.000 ± 0.023	0.563 ± 0.023	0.163 ± 0.021	a/b/c	0.558 ± 0.010	0.271 ± 0.053	a/b/c	0.360 ± 0.011	0.202 ± 0.024	a/b/c
Total aminoacids	1.000 ± 0.333	2.875 ± 0.958	4.547 ± 1.516	a/b/c	2.104 ± 0.701	4.319 ± 1.440	a/b/c	2.649 ± 0.883	4.938 ± 1.646	a/b/c
Glucose	1.000 ± 0.006	0.727 ± 0.006	1.009 ± 0.044	a/b/a	0.620 ± 0.008	0.991 ± 0.025	a/b/a	0.798 ± 0.009	0.758 ± 0.016	a/b/c
Fructose	1.000 ± 0.095	0.929 ± 0.190	1.250 ± 0.038	a/a/a	1.068 ± 0.061	1.804 ± 0.055	a/a/b	1.111 ± 0.123	1.684 ± 0.174	a/a/b
Sucrose	1.000 ± 0.023	0.770 ± 0.110	0.581 ± 0.028	a/b/b	0.692 ± 0.027	0.487 ± 0.016	a/b/c	0.566 ± 0.022	0.482 ± 0.014	a/b/c
Starch	1.000 ± 0.035	0.493 ± 0.075	0.952 ± 0.072	a/b/a	0.502 ± 0.021	0.563 ± 0.010	a/b/b	0.120 ± 0.002	0.783 ± 0.053	a/b/c
Nitrate	1.000 ± 0.106	0.735 ± 0.179	0.179 ± 0.033	a/a/b	0.612 ± 0.072	0.239 ± 0.026	a/b/c	0.664 ± 0.067	0.171 ± 0.023	a/b/c

## Discussion

Here we demonstrate that the isoforms of *PARP* play non-redundant roles in Arabidopsis under non-stressed conditions. Although the metabolic role of *PARP* has been the subject of considerable research in mammals where for example roles have been documented for *PARP1* in mitochondrial metabolism and *PARP2* in pancreatic function and whole body energy expenditure (Bai et al. 2011a, b; Luo et al. 2001), that in plants is much less well characterized. Indeed, until recently such studies were limited to observations of correlative increases in PARP activity, pyridine nucleotide cycling and the nuclear localization of glutathione during exponential cell growth of Arabidopsis (Pellny et al. 2009) and the observation that lines silenced in PARP activity using RNAi exhibited a better energy use efficiency by reducing NAD^+^ breakdown (de Block et al. 2005). More recent studies of PARP function revealed a 30 % increase in NAD^+^ levels and considerable changes in the expression of genes associated with anthocyanin biosynthesis and a strong reduction of anthocyanin accumulation in stress conditions (Schulz et al. 2012). Furthermore a detailed study of the growth in *PARP* inhibited Arabidopsis plants revealed enhanced ATP and NAD^+^ levels but no clear increases in the levels of other primary metabolites leading the authors to conclude that any additional primary metabolites made following treatment were immediately utilized in the support of growth (Schulz et al. 2014). It is, however, important to note that these studies were either indirect or reliant on the use of chemical inhibitor studies or an RNAi approach targeting all PARP isoforms and as such cannot discriminate between the individual roles of the different isoforms of the enzyme. By contrast, a recent study has comprehensively characterized the function of the sirtuin SRT2 which functions as a mitochondrial lysine deacetylase whilst simultaneously producing one molecule of nicotinamide (König et al. 2014). The study suggested the role of SRT2 in the regulation of specific proteins, such as ATP synthase and ATP/ADP carriers and thus its involvement in mitochondrial energy metabolism.

Here we show that the PARPs have a similar yet distinct metabolic function to SRT2. In addition we performed a detailed analysis of the expression profiles of the PARPs as well as studying the localization of their gene products and the distribution of PARP activity. When taken together these combined data reveal novel roles for *PARP* genes under non-stress conditions in addition to the better characterized roles in DNA repair and defense response (Amor et al. 1998; Babiychuk et al. 2001; Berglund and Ohlsson 1995; Rissel et al. 2014; Song et al. 2015) Interestingly, *PARP*1 and *PARP*3 appear to have more important metabolic roles, which is perhaps consistent with the recent observation that PARP2 is the dominant isoform for DNA damage repair and immune responses (Song et al. 2015) That *PARP*3 displays such dramatic metabolic changes in leaves and roots is rather surprising in light of its expression pattern. We cannot at present explain the precise mechanism underlying this, however, two possibilities warrant commenting upon. The first and one we favor is that this effect is somehow carried over from effects in the seed wherein *PARP*3 is massively expressed whilst the second is that modulations of this very low expression is sufficient for the phenotype (providing that the location is correct—see below). It is additionally important to note that changes in the root metabolism of the *PARP*3 mutants could be the consequence of its restricted root growth and thus be indirect effects. That said in the absence of inducible mutants it is very difficult to tease apart the causality of the metabo- to phenotype relationship.

### Expression and localization of the various PARP isoforms and molecular characterization of T-DNA insertional knockdown mutants

The vast majority of work on the roles of PARP in plants have focused on its role in DNA repair or, in the case of the few metabolic studies, have been either indirect (Pellny et al. 2009), or carried out using chemical inhibitors which are likely to inhibit all isoforms to a similar extent (Schulz et al. 2012, 2014). For this reason as a first step to characterize the individual non-redundant roles of the PARP isoforms we evaluated their levels of expression both by the use of promoter GUS fusions (Fig. 1) and by RNA in situ hybridization experiments (Fig. 2). These results suggest that *PARP2* has a distinctive expression pattern from the other isoforms being not or only weakly expressed in seeds but exhibiting high expression in vegetative meristems, whilst *PARP1* was the only isoform expressed in floral tissues and as such suggest at least partially non-redundant functions. However, that said, there is considerable overlap between *PARP1* and *PARP3* expression and all isoforms are expressed in the root where they may play redundant functions. Intriguingly, however, by contrast to a previous report (Boltz et al. 2014), our expression analysis did not support a negative correlation between the expression of PARP1 and PARP2, suggesting that this may well not be a direct effect.

We additionally assessed the subcellular localization of the isoforms using p35S::PARP::GFP fusion constructs (Fig. 3). These data were largely in agreement with sequence based predictions (BAR, bar.utoronto.ca, Toufighi et al. 2005) in suggesting nuclear localizations (although we could not see this for PARP2) but also additional extra-nuclear localizations with signals for PARP1 also present in the chloroplast, PARP2 the chloroplast and mitochondria and PARP3 in the cytosol. Finally, we analyzed the expression of *PARP* genes in the knockdown lines: *parp1*, *parp2* and *parp3* (Fig. 4). A complete lack of expression was observed as expected when analyzing the *PARP* gene in its respective T-DNA line, furthermore a complementary increase of *PARP1* was seen in the *parp*-*3* mutant. We next evaluated the total cellular NAD^+^ hydrolase activity in whole leaf and nuclear extracts of the mutants using an end-point assay determination of nicotinamide production over time. These assays revealed mild yet non-significant decreases of NAD^+^ hydrolase activity in whole leaves of the *parp1* and *parp2* mutants and significant decreases (of up to 50 %), in the nuclear activities in these mutants (Fig. 7), with no change in the level of the *parp*3 mutant consistent with the negligible expression of this isoform in leaf tissue. Interestingly, despite the clear indication of extra-nuclear localization of PARP proteins both from our GFP studies and, at least in the leaf, the NAD^+^ hydrolase activity is clearly predominantly localized in the nucleus. This probably goes some way to explaining why PARPs have not been reported to date in organellar proteome surveys (suba.plantenergy.uwa.edu.au), however, it is important to note that there is plenty of anecdotal evidence that low abundance proteins are below the level of detection of such methods.

### Effect of knockout of PARP isoforms on growth phenotypes

The first obvious phenotype of the *parp* mutants was that all three mutants germinated faster even though the final germination efficiency was invariant from wild type Previous studies have already proposed the relationship of *PARP*s in protecting the seeds from genotoxic stress (Hunt and Gray 2009; Hunt et al. 2007) and although no direct evidence has yet been provided for the involvement of *PARP* in seed dormancy it was recently shown that *PARP3* deficient plants do not maintain germination rates after storage (Rissel et al. 2014). The role of NAD^+^ metabolism in regulating seed dormancy has been much discussed with seed poly(ADP-ribose) levels correlating well with sensitivity of germination to the DNA damaging agent methyl methanesulphonate, whilst the level of NAD^+^ appears to affect the depth of dormancy potentially by enhancing abscisic acid (ABA) synthesis (Hunt and Gray 2009). Consistent with this hypothesis is the finding that genes knocked out in the nicotinamidase gene *NIC*2 display retarded germination and were hypersensitive to addition of nicotinamide or ABA suggesting that parp activity had been impaired (Hunt et al. 2007). Our results suggest that although *parp3* appears to be considerably more highly expressed in the seed than the other isoforms, it has very little influence on the rate of germination. The rate of root elongation was, by contrast, more dramatically altered with all three mutants but with *parp*-2 and *parp*-3 especially displaying considerably accelerated root growth. Results of GC–MS based metabolite profiling, however, revealed that no metabolic changes were observed in all three mutants. Arguably of highest interest is the fact that the photorespiratory metabolites glycerate, glycine, glycolate and serine all decreased in *parp3*). This is of high interest given the clearly established link between NAD^+^ metabolism, photorespiration and nitrogen assimilation in folial tissues (Bauwe et al. 2010) and our recent finding of important metabolic roles for these enzymes in Arabidopsis roots (Nunes-Nesi et al. 2014).

### Effect of downregulation of PARP isoforms on leaf metabolism and function

The canonical marker for chemical PARP inhibition is the accumulation of NAD^+^ (de Block et al. 2005). Interestingly, the level of this metabolite was only significantly increased in leaves of the *parp1* mutant, with the NADH/NAD^+^ ratio even being higher in the *parp3* mutant. Conversely the NADP^+^ and NADPH levels both increased in *parp1* and *parp3* mutants with the NADPH/NADP^+^ ration being unaltered in both mutants. These results thus suggest that the increase in NAD^+^ observed following chemical inhibition of PARP most likely largely reflects a modulation of the PARP1 isoform. A considerable amount of further metabolic changes were observed in the *parp* mutants most notably in *parp1* (which displayed significant alterations in the contents of 15 metabolites) but also in *parp2* and *parp3* (which displayed 14 and 13 changes, respectively). Despite the large degree of change in metabolite levels intriguingly only the increase pyroglutamate was observed in all lines. There are however multiple examples of changes that were conserved between two out of the three lines with *parp1* and *parp3* sharing an additional five changes, whereas *parp1*- and -2 shared an additional three and *parp2* and -*3* shared three additional changes (Fig. 13), with the remaining changes being unique to one of the isoforms. When comparing these changes to those previously reported for other mutants associated with NAD^+^ metabolism (König et al. 2014; Schippers et al. 2008), some interesting commonalities are apparent. For example gluatamine, which was significantly increased in two of the three *parp* mutants was also increased in plants mutated in the parallel sirtuin activity but changes in sugars, organic acids and the other amino acids were dissimilar between the *srt2* mutant and the *parp* mutants (compare data from this study with that from König et al. 2014). The changes in at least *parp1* and *parp3* were however considerably more similar to those in the *old5* genotype which is a mutant of quinolate synthase, a key enzyme in the de novo synthesis of NAD^+^. For example the changes in sucrose, malate and aspartate were highly similar in *parp1*, *parp3* and *old5* (compare data from this study with that from Schippers et al. 2008). Whilst there is considerable overlap between the changes in the various mutants associated with NAD^+^ biosynthesis there was surprisingly little commonality in the *parp* mutants metabolic phenotype and that induced following chemical inhibition by 3-Methoxybenzamide (3 MB) (Schulz et al. 2014). Moreover, the changes in the mutants are quite distinctive from those observed in the roots tending to be quite similar between the lines despite the fact that *PARP3* expression is considerably lower than the other isoforms in this tissue. Indeed the changes are largely consistent with those of the gas exchange measurements which revealed no difference in the rate of photosynthesis but a mild yet insignificant reduction in the rate of respiration. The majority of the changes are similar to those observed previously following the manipulation of mitochondrial metabolism. For example, the up regulation of dehydroascorbate mirrors that observed following the up-regulation of the galacto-1, 4-lactone dehydrogenase donation to the mitochondrial electron transport chain (Nunes-Nesi et al. 2005). Finally, parp-3 roots are characterized by massive and conserved changes in the levels of all detected photorespiratory intermediates this finding is highly interesting particularly given current models of the interaction of NAD^+^ with photorespiration (Bauwe et al. 2010), and our recent findings that the majority of this pathway is fully operational in heterotrophic tissues (Nunes-Nesi et al. 2014).

*In summary*, this study provides the first detailed molecular characterization of the PARP family in Arabidopsis and comprehensively characterizes the non-nuclear metabolic and physiological role of the independent isoforms under non-stress conditions. These studies provide support for current models of the linkage between NAD^+^ metabolism, the TCA cycle and associated mitochondrial metabolism and photorespiration revealing an role for them in the co-ordination of central metabolism. Given that the metabolic consequences of PARP deficiency were quite distinctive from that following the inhibition of the parallel reaction catalyzed by sirtuin (König et al. 2014), it is apparent that these proteins have differential roles in plant function with the PARPs seemingly more important with regard to metabolism. It is additionally intriguing that there is a significant increase in the levels of NADP(H) in the leaves of Parp1 and -3 mutants whilst PARP would have been anticipated to rather effect NAD^+^ levels. In this context it is additionally interesting to compare and contrast our data with that found on the overexpression of NAD^+^ kinase in rice and Arabidopsis (Takahara et al. 2010; Takahashi et al. 2009). The NAD^+^ kinase also had only marginal effects on NAD^+^ levels but considerably elevated NADP(H) levels and were characterized by elevated amino acid content but little change in the levels of most other metabolites measured here. These results are partially overlapping with those of the PARP1 and PARP3 mutants i.e. for those amino acids which increase although it is important to note that many of the changes are opposite. Thus studies looking at the role of NADP(H) in the regulation of the commonly changing amino acids would likely be a promising avenue for future research. Finally, the root and seed phenotypes of the *parp* mutants provide direct proof for the previous postulates of a role for PARP in these developmental processes with our data suggesting a possible link between enzymes of the photorespiratory pathway and root growth. We are thus able to conclude that *PARP* isoforms play partially overlapping but non-functionally redundant roles in Arabidopsis metabolism and have importance above and beyond that already inferred by testing their role with regard to oxidative stress. It seems likely that organelle specific overexpression of PARP activities would be a highly informative approach by which to gather further understanding of their compartment specific functions and should be a priority for future studies.

## Materials and methods

### Chemicals

All standard chemicals and enzymes were obtained from Fluka (Seelze, Germany), Merck (Darmstadt, Germany), Roche (Mannheim, Germany) and Sigma Aldrich (Deisenhofen, Germany), unless stated otherwise.

### Plant material

All *Arabidopsis thaliana* plants used in this study were of the *Columbia* ecotype (Col-0). The *poly(ADP*-*ribose)polymerase* (*PARP*) T-DNA mutant lines *parp1* (SAIL_632_D07), *parp2* (SALK_111410) and *parp3* (SAIL_1250_B03) were obtained from Nottingham Arabidopsis Stock Centre (University of Nottingham, UK). Homozygous mutant lines were isolated by PCR using primers specific for *PARP1* (P1-N878-LP, 5′-ACTTAATTGTGGTTTGACGCC-3′ and P1-N878-BP, 5′-CCGTCTTCTAGTGTTTGAGCC-3′) in combination with the T-DNA left border primer (SAIL-LB 3.1, 5′-CATCTGAATTTCATAACCAATCTCG-3′) for *parp1*, or primers specific for *PARP2* (P2-N611-LP, 5′-AATTAAAGATGGACATTCGCG-3′ and P2-N611-RP, 5′-CAGAGAGAACAGGATTGAACCC-3′) in combination with the T-DNA left border primer (SALK-LBb 1.3, 5′-ATTTTGCCGATTTCGGAAC-3′) for *parp2*, and primers specific for *PARP3* (P3-N875-LP, 5′-GTGGTACGTCAAAGATGGTGG-3′ and P1-N878-BP, 5′-TTGCAACAAACGAATCAAGC-3′) in combination with the T-DNA left border primer (SAIL-LB 3.1) for *parp3.*

Seeds were surface-sterilized and imbibed for 2 days at 4 °C in the dark prior to plating on half-strength Murashige and Skoog (MS) media with 1 % sucrose. Seeds were subsequently germinated and grown at 20 °C under long-day conditions (16 h light/8 h dark) with 150 μmol m^−2^ s^−1^. For germination rate and root growth analysis seeds were sown on MS media with 1 % sucrose.

### GUS reporter activity assay

DNA fragments of the *AtPARP* promoters were amplified from the genomic DNA of Col-0 using the primers 5′-CACCTAGACGTGTGTAAAGTAGGCAAAGTG-3′ and 5′-TTTCGTCTTCTTCTTCAGGAGAATAG-3′, 5′-CACCTTTATTCACCATTTCTCTGCTTCTC-3′ and 5′-TTCTCCGGTAAGAGACAATTACACA-3′, or 5′-CACCATCGGTAAATGTAGCCAATAAGAC-3′ and 5′-TGAGCAAACTCTTTGAACTGTATG-3′ for *PARP1*, *PARP2* or *PARP3,* respectively. The PCR product was recombined into pENTR/SD/D/TOPO (Invitrogen) and subsequently transferred into pKGWFS7 vector using Gateway technology. Transformants were selected with 50 mg L^−1^ kanamycin and GUS activity was detected by stereo electron microscope (Leica MZ12.5 stereomicroscope with Leica DFC420 digital camera) after vacuum infiltration with 2 mM 5-bromo-4-chloro-3-indolyl-*β*-d-Glucuronide in 50 mM sodium phosphate (pH 7.0), containing 0.2 % TritonX-100. Samples were incubated overnight at 37 °C and cleared in 70 % ethanol.

### Assaying GFP expression in protoplasts

Full length coding sequences of *PARP* genes was isolated from Col-0 using the primers 5′-CACCATGGCGAACAAGCTCAAAGTC-3′ and 5′-GTGCTTGTAGTTGAATTTGACTTGG-3′, 5′-CACCATGGCAAGCCCACATAAGC-3′ and 5′-TCTCTTGTGCTTAAACCTTACTTTC-3′, or 5′-CACCATGAAGGTTCACGAGACAAGATC-3′ and 5′-CTCTGGTTCGACATCGACTATC-3′ for *PARP1*, *PARP2* or *PARP3* respectively. The PCR product was recombined into pENTR/SD/D/TOPO (Invitrogen) and subsequently transferred into pK7FWG2 vector using Gateway technology. Transformants were selected with 50 mg L^−1^ kanamycin. Plasmid construct p35S::NLS::GFP was used as control for nuclear localization. Protoplast isolation was performed by the TAPE-*Arabidopsis*-Sandwich protocol (described in Wu et al. 2009). Subcellular localization of PARP in plant compartments was detected under laser scanning confocal microscope (Leica TCS SP5) through fluorescence of GFP (green), mitotracker (blue) as well as chlorophyll auto—fluorescence (red).

### RNA in situ hybridization

Plants were grown under long day (16 h light/8 h dark, 150 μmol m^−2^ s^−1^) at 22 °C and samples were collected from vegetative, reproductive plants and various embryonic stages. Fixation, dehydration and embedding into wax as well as probes synthesis and RNA in situ hybridization were performed as described in (Wahl et al. 2013). Histological sections were imaged with an Olympus BX-61 microscope equipped with a DC View III digital camera.

### NAD hydrolase enzymatic assay

The assay was carried out with two different sets of extracts (nuclei only extract and whole cell extracts). The preparation of isolation buffer (consisting of 2 M hexylene glycol; 20 mM PIPES-KOH, pH 7.0; 10 mM MgCl_2_ and 5 mM *β*-mercaptoethanol) was prepared (exactly as described Folta and Kaufman 2006). Biological replicates of 0.5 g fresh Arabidopsis rosette leaves were harvested and grind in 10 mL of isolation buffer before filtering through two layers of miracloth. The filtered suspension was supplemented with 2 mL lysis buffer containing 10 % Triton-X100 in isolation buffer (for nuclei extract) and shook gently on ice or kept on ice in regards of whole cell extracts. After seven min the falcons were centrifuged at 4 °C for 10 min at 2000 g. Lastly, the pellet was dissolved in 1 mL storage buffer (consisting of 50 mM Tris–HCl, pH 7.8; 20 % glycerol; 5 mM MgCl_2_; 0.44 M sucrose and 10 mM *β*-mercaptoethanol). PARP activity assay was performed as described in (Tian et al. 1999). For each cell suspension and genotype, blank assay without addition of substrate was included. In parallel to the samples, nicotinamide standards (100–500 ng to 1–2 μg) were prepared and reduced to dryness in a Speedvac^®^ overnight after addition of 60 μL ribitol. Extracted and dried samples above were extracted another time as well as derivatized with the protocol for metabolic profiling. Samples were put to run on the gas chromatography-mass spectrometry (GC–MS) with the nicotinamide (NaM)-standards, an Arabidopsis control and one blank control. The enzyme activities of *PARP* genes were determined by the amount of NaM via GC–MS method normalized to blank assay, internal standard, g fresh weight and incubation time.

### Measurements of photosynthetic parameters

Leaf gas-exchange measurements were performed with an open-flow gas exchange system Li-Cor 6400 (Li-Cor Inc., Lincoln, NE, USA). Three-week-old plants grown in short day conditions and before bolting were used for determination of the photosynthetic parameters. Light response curves were carried out by varying the photosynthetic photon flux density (PPFD) from 1000 μmol m^−2^ s^−1^ to zero. The reference CO_2_ concentration was set at 400 μmol CO_2_ mol^−1^ air. All measurements were performed at 25 °C and the amount of blue light was set to 10 % PFD to optimize stomatal aperture. Additionally, using a dark-adaptation leaf clip, minimum (*F*_0_) and maximum (*F*_m_) dark-adapted (30 min) fluorescence were measured, from which *F*_v_/*F*_m_ ratio, in which *F*_v_ = *F*_m_ − *F*_0_, was calculated. This ratio has been used as a measure of the potential photochemical efficiency of PSII.

### Measurement of respiratory parameters

Dark respiration was measured using the same gas exchange system as defined above by adapting plants for 30 min in the dark to avoid light-enhanced dark respiration. Estimations of the TCA cycle flux on the basis of ^14^CO_2_ evolution were carried out following incubation of isolated leaf discs in 10 mM MES-KOH, pH 6.5, containing 2.32 KBq mL^−1^ of [1-^14^C]-, [2-^14^C]-,[3,4-^14^C]-, or [6-^14^C]-glucose. ^14^CO_2_ evolved was trapped in KOH and quantified by liquid scintillation counting. The results were interpreted following Ap Rees and Beevers (1960).

The ^14^C-labeling pattern of sucrose, starch, and other cellular compounds was determined by incubation of isolated leaf discs in 10 mM MES-KOH, pH 6.5, containing 10 μCi of [U-^14^C]-glucose at a PFD of 700 μmol m^−2^ s^−1^ in 22 °C for 2 h and subsequent fractionation was performed exactly as defined by Lytovchenko et al. (2002).

### Metabolic profiling

Four-week-old plants were harvested for metabolite analysis. Chlorophyll, sucrose, starch, total protein, total amino acid and nitrate contents were determined as described in (Sienkiewicz-Porzucek et al. 2010), while NAD(H) and NADP(H) were determined as described previously (Schippers et al. 2008). Metabolite extraction for GC–MS was performed on the same samples as used for metabolite determination by spectrophotometric methods. The extraction, derivatization, standard addition, and sample injection and machine used were exactly as described by Lisec et al. (2006). Target metabolites were annotated using Chroma TOF 1.0 (Leco, http://www.leco.com/) and TagFinder 4.0 software on the basis of exact retention times and their corresponding mass spectra (Lüdemann et al. 2008). Full documentation of metabolite profiling data acquisition and interpretation is provided in supplemental dataset (Tables. S1 and S2) online following recommended guidelines (Fernie et al. 2011).

### RNA extraction and qRT-PCR analysis of gene expression

Quantitative Real Time PCR was carried out exactly as described in Czechowski et al. (2004). (Czechowski et al. 2004). Primers used here are described in the Table S3. RNA was extracted from at least five biological replicates Extraction of total RNA from leaves, flowers and siliques was performed using TRIzol reagent (Invitrogen); from roots as described in Bugos et al. (1995) with minor modifications; and from seeds as described in Birtic and Kranner (2006). Digestion with DNAse I (Ambion) was performed according to the manufacturer’s instructions. To confirm the absence of genomic DNA contamination, a quantitative PCR analysis using specific primer pairs was performed. The primer pairs were designed to amplify intron sequence of *Actin2* (*At3g18780*, *ACT2* forward 5′-ACTTTCATCAGCCGTTTTGA-3′ and reverse 5′-ACGATTGGTTGAATATCATCAG-3′). The integrity of the RNA was checked on 1 % (w/v) agarose gels, and the concentration was measured before and after DNase I digestion using a Nanodrop ND-1000 spectrophotometer. cDNA was synthesized from 2 μg total RNA using Superscript III reverse transcriptase (Invitrogen) according to the manufacturer’s instructions. The efficiency of cDNA synthesis was estimated by quantitative PCR using two primer pairs amplifying the 5′ and 3′ regions of glyceraldehyde 3-phosphate dehydrogenase (*GAPDH*5′, forward primer 5′-TCTCGATCTCAATTTCGCAAAA-3′/reverse primer 5′-CGAAACCGTTGATTCCGATTC-3′ and *GAPDH*3′ forward 5′-TTGGTGACAACAGGTCAAGCA-3′/reverse primer 5′-AAACTTGTCGCTCAATGCAATC-3′). The PCR reaction was performed in a 5 μL volume as described by Caldana et al. (2007). Data analysis was performed using SDS software version 2.4 (Applied Biosystems). Genes expressions were normalized against the constitutively expressed ubiquitin (*UQ*) using the following primer pair 5´-AGCAGTTGGAGGATGGCAGAAC-3´ and 5´-CGGAGCCTGAGAACAAGATGAAGG-3′.

### Statistical analysis

The term significant is used here only when the change in question has been confirmed to be significant (*Pp* < 0.05 or *Pp* < 0.01) with the Student’s *t* test. All statistical analyses were performed using the algorithm embedded into Microsoft Excel.

## Electronic supplementary material

Supplementary material 1 (PPTX 543 kb)

Supplementary material 2 (XLSX 27 kb)
