# Supplementary material for: Analysis of knockout mutants reveals non-redundant functions of poly(ADP-ribose)polymerase isoforms in Arabidopsis
Source: Plant Mol Biol. 2015 Oct 1;89(4-5):319–38. doi: 10.1007/s11103-015-0363-5 (PMC4631723; doi:10.1007/s11103-015-0363-5)
Supplement: Supplementary file 1 — Supplementary material 1 (PPTX 543 kb) [file 11103_2015_363_MOESM1_ESM.pptx]

## Slide 1
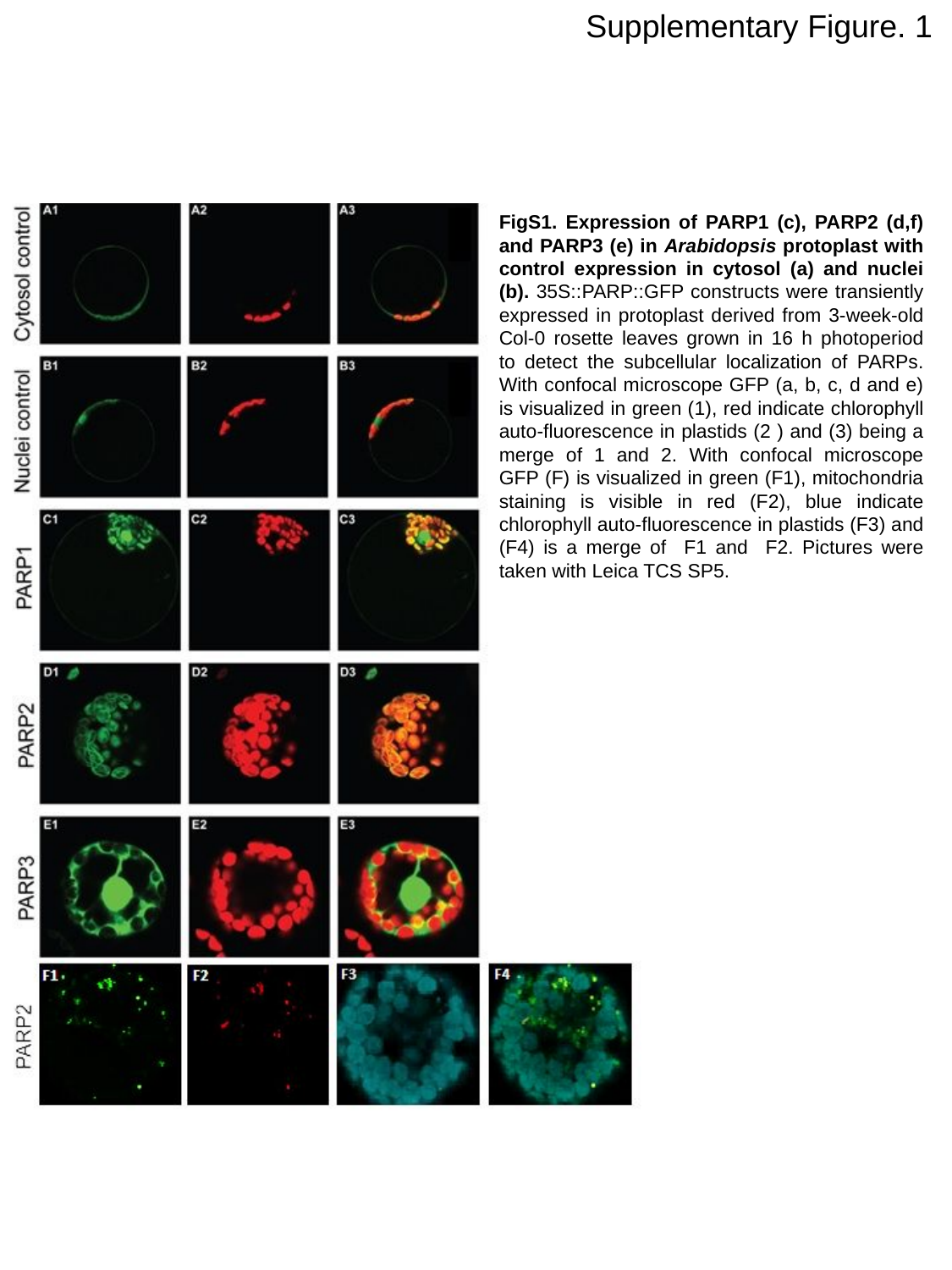

Supplementary Figure. 1
FigS1. Expression of PARP1 (c), PARP2 (d,f) and PARP3 (e) in Arabidopsis protoplast with control expression in cytosol (a) and nuclei (b). 35S::PARP::GFP constructs were transiently expressed in protoplast derived from 3-week-old Col-0 rosette leaves grown in 16 h photoperiod to detect the subcellular localization of PARPs. With confocal microscope GFP (a, b, c, d and e) is visualized in green (1), red indicate chlorophyll auto-fluorescence in plastids (2 ) and (3) being a merge of 1 and 2. With confocal microscope GFP (F) is visualized in green (F1), mitochondria staining is visible in red (F2), blue indicate chlorophyll auto-fluorescence in plastids (F3) and (F4) is a merge of F1 and F2. Pictures were taken with Leica TCS SP5.
